# Preliminary testing of the reliability and feasibility of SAGE: a system to measure and score engagement with and use of research in health policies and programs

**DOI:** 10.1186/s13012-017-0676-7

**Published:** 2017-12-19

**Authors:** Steve R. Makkar, Anna Williamson, Catherine D’Este, Sally Redman

**Affiliations:** 1 The Sax Institute, Level 13, Building 10, 235 Jones Street, Ultimo, New South Wales 2007 Australia; 2 National Centre for Epidemiology and Population Health (NCEPH), Research School of Population Health, The Australian National University, 62 Mills Road, Acton, Australian Capital Territory 0200 Australia

## Abstract

**Background:**

Few measures of research use in health policymaking are available, and the reliability of such measures has yet to be evaluated. A new measure called the Staff Assessment of Engagement with Evidence (SAGE) incorporates an interview that explores policymakers’ research use within discrete policy documents and a scoring tool that quantifies the extent of policymakers’ research use based on the interview transcript and analysis of the policy document itself. We aimed to conduct a preliminary investigation of the usability, sensitivity, and reliability of the scoring tool in measuring research use by policymakers.

**Methods:**

Nine experts in health policy research and two independent coders were recruited. Each expert used the scoring tool to rate a random selection of 20 interview transcripts, and each independent coder rated 60 transcripts. The distribution of scores among experts was examined, and then, interrater reliability was tested within and between the experts and independent coders. Average- and single-measure reliability coefficients were computed for each SAGE subscales.

**Results:**

Experts’ scores ranged from the limited to extensive scoring bracket for all subscales. Experts as a group also exhibited at least a fair level of interrater agreement across all subscales. Single-measure reliability was at least fair except for three subscales: Relevance Appraisal, Conceptual Use, and Instrumental Use. Average- and single-measure reliability among independent coders was good to excellent for all subscales. Finally, reliability between experts and independent coders was fair to excellent for all subscales.

**Conclusions:**

Among experts, the scoring tool was comprehensible, usable, and sensitive to discriminate between documents with varying degrees of research use. Secondly, the scoring tool yielded scores with good reliability among the independent coders. There was greater variability among experts, although as a group, the tool was fairly reliable. The alignment between experts’ and independent coders’ ratings indicates that the independent coders were scoring in a manner comparable to health policy research experts. If the present findings are replicated in a larger sample, end users (e.g. policy agency staff) could potentially be trained to use SAGE to reliably score research use within their agencies, which would provide a cost-effective and time-efficient approach to utilising this measure in practice.

**Electronic supplementary material:**

The online version of this article (10.1186/s13012-017-0676-7) contains supplementary material, which is available to authorized users.

## Background

In order to improve the use of research in health policymaking, policy agencies globally have begun implementing capacity-building strategies such as providing training programs to improve staff skills in accessing, appraising, and applying research to policy [1] or building technical infrastructure to support research access and use among staff [2]. These capacity-building efforts could be strengthened, or better targeted, if agencies were able to measure whether and how staff currently engage with and use research to inform their policies and programs [3]. However, only six such measures are currently available that assess research use in health policymaking [4–11]. These measures have limitations such as exclusively focusing on direct (i.e. *instrumental*) use of research in policy [7–9, 11], lack of a clear theoretical basis [6, 12], not being targeted at specific policies or programs [4–9], and absence of a systematic system to score research use [4, 10].

To overcome these limitations, a comprehensive measure called SAGE (Staff Assessment of Engagement with Evidence) has been developed [13–15]. It consists of a semi-structured interview with a policymaker about how research was *engaged with* and *used* in a recently developed policy document. The empirical development of the SAGE interview is described elsewhere [16]. SAGE is heavily based on the Supporting Policy in Health with Research: an Intervention Trial (SPIRIT) Action Framework [17–22], a theoretical model grounded in organisational theory and conceptual models of evidence-based decision-making, which summarises the hypothesised relationship between research and policy formulation. The SAGE interview focuses on two key aspects of the framework: (A) research engagement actions and (B) research use. Research engagement actions refer to actions that enable policymakers to access and/or generate relevant research to inform policy formulation [17] and include (1) searching for and (2) obtaining research, (3) appraising its relevance to the policy issue and (4) its quality in terms of methodological rigour and validity, (5) generating new research and/or data analyses (especially when research on the current policy issue is not available), and (6) interactions with researchers. Consequently, in SAGE, interviewees are asked to describe how they searched for, appraised, and generated research and interacted with researchers during the process of developing the policy document in question.

According to the framework, if the policymaker performs one or more of these research engagement actions, research can then be (B) used to inform policy in one of four ways: (1) to directly inform decisions relating to the identified policy issue(s) (instrumental use; [15, 23, 24]), (2) to clarify understanding of the policy issue without directly influencing decisions (conceptual use [25–27]), (3) justify and/or persuade others to support a predetermined decision (tactical use [28, 29]), or (4) to meet legislative, funding, or organisational requirements (imposed use [30]). Based on this multifaceted definition of research use, SAGE invites interviewees to describe whether research was used to directly inform policy direction and content (i.e. instrumental use), increase their understanding of policy issues (i.e. conceptual use), persuade others to support pre-existing policy decisions (i.e. tactical use), and/or to satisfy their agency’s requirements to use research (i.e. imposed use). As such, SAGE assesses research use beyond direct instrumental use. The SPIRIT Action framework predicts that when research is used to inform health policies and programs, this will ultimately lead to better health services and outcomes.

SAGE is accompanied by a systematic scoring tool, which scores the extent to which policymakers undertook research engagement actions and used research, based on their interview responses. The scoring tool breaks down each research engagement action and type of research use into smaller key actions (called *subactions*). Each subaction has a point value assigned to it reflecting its importance for achieving evidence-informed health policy, based on the opinions of over 50 experts in health policy and research [13, 14]. The points for all subactions performed by a policymaker are summed to yield a total score for the research engagement action or research use type being measured.

Having developed the SAGE interview and empirically established a system of scoring responses in the interview, the aim of the present study was to provide preliminary evidence of the feasibility, sensitivity, and reliability of the SAGE scoring tool. This is important given the relative absence of reliable, thorough, and practical tools that measure how research is engaged with and used to shape health policy decisions within agencies. To do this, we recruited researchers with expertise in the health policy and research nexus to examine whether the SAGE scoring tool was acceptable and comprehensible (e.g. does it make sense?), whether the measure yielded scores across all points of the scale (e.g. can the scoring tool discriminate between documents where research was used more versus less?), and whether the scoring tool achieved a sufficient level of interrater agreement (i.e. is it reliable?). This sample was chosen because of their extensive understanding of health research and policy and the contextual factors that impact the use of research in policymaking. Because of this expertise, their ratings were considered ‘the gold standard’ —that is, the best approximation to policymakers’ true research engagement and use scores on SAGE.

To more directly test the reliability and practical utility of the scoring tool, we recruited independent coders who were not experts in health policy research, to use the SAGE scoring tool to rate policy documents. We examined whether their ratings agreed with each other and with those made by the health policy researchers. This would not only further test the reliability of the SAGE scoring tool, but provide an indication of its practical utility. Specifically, it would be more time-efficient and cost-effective to recruit coders (e.g. policymakers, agency staff, end users) who are not necessarily health policy research experts to score SAGE within agencies, as opposed to recruiting outside experts.

## Methods

### Participants

We recruited two types of participants: (i) experts and (ii) independent coders. Experts were defined as researchers who had published widely in evidence-informed health policymaking (i.e. a field that examines how research evidence is used to shape health policy decisions and programs [31, 32]), held senior positions in their respective organisations (e.g. at least associate professor level if working at a university or equivalent), and worked in Australia or countries with comparable health systems (e.g. the UK, Canada). We selected these experts because of their presumed extensive knowledge of the health policy landscape, health research, the contextual factors that influence whether or not research is used in policy, and a firm understanding of the domains being scored in SAGE (e.g. instrumental use, tactical use). Experts were recruited from members within the CIPHER (Centre for Informing Policy in Health with Evidence from Research) community, a federally funded Centre of Research Excellence which aims to improve policy agencies’ capacity to use research in health policy formulation [17].

Independent coders were eligible to participate if they possessed Masters-level qualifications in a relevant field (e.g. public health), but were not researchers in the field of evidence-informed policy. All potential participants were sent an invitation email detailing the background and aims of the study. The experts were contacted directly by the study’s chief investigator (SR). For the independent coders, an internal email was sent to all Sax Institute staff describing the study and roles of coders. Interested individuals were invited to send a reply email to the first author confirming their interest in participating in the study. Those that expressed interest were sent an information sheet and consent form providing further detailed information on the aims, background, and methods of the study, as well as privacy, remuneration, and conflict of interest.

### Materials

#### SAGE interview

The SAGE interview comprises a series of open-ended questions that ask the policymaker to describe the following:

1. Whether or not research was used to inform that document’s development
2. How research was (1) searched for, (2) what research was obtained, how that research was appraised for (3) relevance and (4) quality, (5) whether new research or data analyses were generated, and (6) what sorts of interactions with researchers occurred, if any (i.e. *research engagement actions*)
3. If/how research informed the development of the document (i.e. *types of research use*—(1) conceptually, (2) instrumentally, (3) tactically, and (4) imposed)
4. Any barriers and facilitators that influenced their capacity to use research.

Interviews were audio recorded and professionally transcribed. The interviewer checked, corrected, and de-identified transcripts before they were scored. The full interview is provided in Additional file 1.

#### SAGE customised interview transcripts

Sixty (*n* = 60) customised interview transcripts were used to assess interrater reliability of the SAGE scoring tool. The original transcripts came from SAGE interviews with policymakers from six Australian health policy agencies participating in an intervention trial known as SPIRIT (Supporting Policy In health with Research: an Intervention Trial) [33]. These original transcripts were read in full by the first author and then rearranged to form *customised transcripts*, whereby text from the original interview was copied and pasted into the relevant sections of a customised transcript template (see Additional file 2). The template contained sections for all six research engagement actions and four types of research use. Questions in the original interview directly aligned with these sections. If interviewees did not provide a response to an interview question that matched a template section, then it was left blank and treated as missing data for that domain. Transcripts were customised to streamline the scoring process due to the excessive length of the original transcripts.

When the original transcripts contained text about particular research engagement or research use actions *outside* of the questions directly addressing that action, this text was copied and pasted into the appropriate section within the customised transcript template. The detailed definitions of each research engagement action and research use type provided in the SAGE scoring tool and instruction booklet (see below) helped guide the search for these details. For example, if an interviewee described study issues such as sample size, research design, or statistical analyses when asked about research relevance (question 9), then based on the definitions within the SAGE scoring tool, this text would be coded as describing research *quality* rather than relevance, and pasted into the *Quality Appraisal* section of the template.

#### SAGE scoring tool

The scoring tool is a checklist used to score the extent to which policymakers had undertaken the six research engagement actions and four types of research use (i.e. the SAGE domains). The checklist items are the key subactions of each measured domain. Subactions refer to concrete examples of undertaking each research engagement and research use action. If the rater believes the policymaker undertook a key subaction based on his or her interview, it is ticked off on the checklist.

Each subaction has a different point value assigned to it based on its importance in facilitating evidence-informed health policymaking. The degree of importance of each subaction was established through conjoint analysis of surveys completed by over 50 local and international health policy research experts [13, 14]. The conjoint analysis generated numerical weights for each subaction. Subactions with greater weights were regarded by the experts (on average) as being relatively more important for achieving evidence-informed health policy. All subaction weights within a domain were rescaled so that they added up to a total score of 9 for each domain. The rescaled weight was then used as the final point value for that subaction in the SAGE scoring tool.

The points for all ticked subactions in the scoring tool are summed to give a score for that particular domain. For example, if a policymaker searched academic databases (score = 2.83) and grey literature sources (score = 1.42), they received a score of 4.25 out of 9 for domain A1: *Searching for Research*. Each of the ten measured domains receives a score from 0 to 9, where 1–3 indicates limited, 4–6 moderate, and 7–9 extensive engagement with or use of research. This scoring checklist is presented as a scorecard in Microsoft Excel, which allows raters to select YES or NO from a drop-down menu to rate whether or not a subaction was performed by the policymaker (see Additional file 3).

#### Policy document

The actual policy document and/or the accompanying reference list was provided to raters to supplement the customised transcript. Because we adopted a liberal definition of *policy document* [34], there was quite a wide range of documents, including reviews, reports, discussion papers, formal directives, program plans, strategic plans, ministerial briefs, budget bids, guidelines, evaluations, and resourcing plans. Sixty such policy documents corresponding to the 60 transcripts were used.

#### Instruction booklet

The instruction booklet contained detailed information about the study including background, the full SAGE interview and scoring tool, an outline of the study protocol, and detailed instructions on how to use the scoring tool (see Additional file 4).

#### Design

Based on published interrater reliability guidelines [35–38], a minimum of 51 documents, rated by five coders would achieve a reliability of 0.7 with a confidence interval half-width of 0.2. We thus selected 60 documents to be rated. Because of experts’ busy schedules, it was not feasible for them to score all 60 documents. Consequently, each document was rated by three randomly selected experts and both independent coders. Experts were randomly assigned to documents using a balanced incomplete block design (BIBD) generated in *R*, which ensured each expert rated a total of 20 documents [39–41].

#### Choice of reliability coefficient

We used the reliability coefficient, *G(q,k)* [42, 43], which is appropriate in experimental designs that are neither fully crossed (i.e. where every document is scored by all raters) nor fully nested (i.e. where each document is scored by a different set of raters) as in the present study [36, 42]. *G* is the reliability level and reflects the proportion of the expected score variance that is attributable to true score variance and ranges from 0 to 1, with higher scores indicating greater reliability; *k* refers to the number of raters that rated each document; and *q* is a multiplier that accounts for the degree of rater overlap between documents. The value of *q* approaches 0 as the overlap in raters between documents increases. The interpretation of *G* is akin to the intraclass correlation coefficient [36], so we applied similar cutoff values to evaluate the degree of interrater reliability in the present study: < 0.4 = poor, 0.4–0.59 = fair, 0.6–0.74 = good, and ≥ .75 = excellent.

### Procedure

After participants consented to participate in the study, they were sent the study materials. This included the customised transcripts and accompanying policy documents, the SAGE scoring tool, Excel scorecard, and the instruction booklet. Each expert received 20 out of the 60 transcripts. The two independent coders received all 60 transcripts.

Participants were instructed to first read the Instruction Booklet to ensure they understood how to score each interview transcript. Participants then proceeded to score each transcript. They were encouraged to proceed through the customised transcript and scoring tool sequentially by domain, using the scoring tool to tick off the subactions performed by the policymaker based on his or her responses in the transcript. These steps were repeated for the remaining nine domains in the transcript and scoring tool. After all the ratings were collected for all documents, we computed the total scores for each domain by adding the points for ticked subactions within that domain.

### Data analysis

Based on the study aims, we first examined the range of scores (i.e. minimum and maximum) obtained by experts to determine if they were able to use the scoring tool to discriminate between documents on each measured domain. This would address our first key question regarding whether the SAGE scoring tool was acceptable, comprehensible, and sufficiently sensitive to capture differences between policy documents in the extent to which research was engaged with and used to inform its development.

To address the second major question regarding whether the SAGE scoring tool was reliable and whether independent coders produce comparable scores to experts in the field of evidence-informed health policy (thus providing initial evidence for its practical utility), we tested interrater reliability between the (A) nine experts, (B) the two independent coders, (C) and between experts and independent coders. *G*(*q*, k) coefficients were calculated in SAS. *Single-measure G(q,*1*)* and *average-measure G*(*q*, k) reliability were estimated for both (A) and (B). Single-measure reliability indicates how reliable a single rater—either an expert or independent coder—would be if he or she had undertaken the ratings on his or her own. Average-measure reliability on the other hand, indicates the reliability of the averaged ratings of the experts or the independent coders. To calculate (C), we first averaged experts’ scores for each document. We then calculated single-measure reliability, *G*(*q,*1*)*, as this would demonstrate whether the ratings made by experts could be generalised to the ratings made by a single-independent coder.

## Results

### Participants

Nine (*n* = 9) experts and two (*n =* 2) independent coders participated.

### Use of the SAGE scoring tool among experts

There was no missing data. Table 1 displays the minimum, maximum, and mean scores for the 10 domains as rated by experts (averaged across documents). Scores ranged from 0 to at least 7.76 for all domains when averaged across all nine experts. Therefore, averaged across experts, document scores ranged from limited (i.e. between 0 and 2.99) to extensive (6–9) for all ten domains.

**Table 1:** Means, standard deviations, medians, minimum, and maximum scores on each domain averaged across expert raters

Domain	M	SD	Median	Min	Max
Searching	4.27	2.31	4.25	0.00	9.00
Research obtained	4.54	2.34	4.36	0.00	9.00
Relevance appraisal	3.17	2.05	2.06	0.00	7.76
Quality appraisal	2.50	2.31	2.00	0.00	9.00
Generating new research	2.50	3.23	0.18	0.00	9.00
Interacting with researchers	2.51	3.11	0.49	0.00	9.00
Conceptual research use	3.81	2.82	3.03	0.00	9.00
Instrumental research use	4.12	3.14	4.75	0.00	9.00
Tactical research use	5.79	3.51	6.09	0.00	9.00
Imposed research use	3.60	2.95	5.07	0.00	9.00
Total	3.68	2.98	3.48	0.00	9.00

Additional file 5 displays the minimum and maximum ratings provided by each expert across all ten domains. For six of the domains, all nine experts gave scores that ranged from limited to extensive. These domains were Searching for Research, Research Obtained, Conceptual Use, Instrumental Use, and Tactical Use. For the domains Relevance Appraisal, Interacting with Researchers, and Generating New Research, 7 of 9 raters’ scores encompassed the limited to extensive range, whereas the remainder ranged from limited to moderate. Finally, for Quality Appraisal and Imposed Use, 6 of 9 raters’ scores ranged from limited to extensive, whereas the remaining 3 raters’ scores ranged from limited to moderate.

Analysis of histograms for each domain showed that document scores were normally distributed for five domains (Searching for research, Research Obtained, Relevance Appraisal, Conceptual Use, and Instrumental Use; see Additional file 5). Scores were positively skewed for four domains: Quality Appraisal, Generating Research, Interacting with Researchers, and Imposed Use. For the latter three domains, this occurred because numerous documents attracted a zero score since policymakers reported that no research was generated, no interactions occurred, or research use was not imposed by the organisation.

Figures S1 and S2 in Additional file 6 show the mean scores for each expert for research engagement actions and research use actions, respectively. For research engagement actions, experts generally tended to score Searching for Research and Research Obtained higher than Relevance Appraisal, Quality Appraisal, Generating New Research, and Interacting with Researchers. However, this pattern did not clearly emerge for expert 8. For research use actions, Tactical Use was generally scored higher than Instrumental, Conceptual, and Imposed Use, although expert 2 tended to score all types of research use in the low to moderate range. These results suggest that some experts may have pursued idiosyncratic scoring rules.

#### Reliability between the experts

Table 2 displays single-measure reliability coefficients, and Table 3 displays average-measure reliability for all 10 SAGE domains. As shown in Table 3, average-measure reliability among the experts was excellent (i.e. *G*(*q*, k) ≥ 0.75) for three domains: Searching for Research, Research Obtained, and Imposed Use. Reliability was good (i.e. *G*(*q*, k) values between 0.6 and 0.74) for four domains: Quality Appraisal, Generating New Research, Interacting with Researchers, and Tactical Use. Reliability was fair (i.e. *G*(*q*, k) values between 0.4 and 0.59) for the remaining domains: Relevance Appraisal, Conceptual Use, and Instrumental Use. For single-measure reliability, Table 2 shows that it was good for two domains: Research Obtained and Imposed Use. Reliability was fair for five domains including Searching for Research, Quality Appraisal, Generating New Research, Interacting with Researchers, and Tactical Use. However, reliability was poor (i.e. *G*(*q*, k) values < 0.4) for Relevance Appraisal, Conceptual Use, and Instrumental Use.

**Table 2:** Single-measure reliability coefficients for independent coders, experts, and between independent coders and experts

Aim	Single-measure reliability *G(q,*1*)*									
Domain	Searching for research	Research obtained	Relevance appraisal	Quality appraisal	Generating new research	Interacting with researchers	Conceptual research use	Instrumental research use	Tactical research use	Imposed research use
(A) Reliability between the two independent coders; *q* = 0	0.74	0.78	0.58	0.78	0.86	0.73	0.62	0.62	0.74	0.76
(B) Reliability between the nine experts; *q* = 0.08;	0.57	0.63	0.25	0.50	0.41	0.40	0.35	0.25	0.47	0.62
(C) Reliability between the independent coders and the average of the experts; *q* = 0	0.75	0.69	0.48	0.76	0.73	0.64	0.55	0.57	0.70	0.76

**Table 3:** Average-measure reliability coefficients for independent coders and experts

Aim	Average-measure reliability *G(q,*k*)*									
Domain	Searching for research	Research obtained	Relevance appraisal	Quality appraisal	Generating new research	Interacting with researchers	Conceptual research use	Instrumental research use	Tactical research use	Imposed research use
(A) Reliability between the two independent coders; *q* = 0, *k* = 2	0.85	0.88	0.74	0.88	0.93	0.84	0.76	0.77	0.85	0.87
(B) Reliability between the nine experts; *q =* 0.23, *k* = 3	0.77	0.83	0.46	0.74	0.66	0.65	0.58	0.49	0.71	0.89

#### Reliability between the two independent coders

As shown in Table 3, average-measure reliability for the two independent coders was excellent for all domains (i.e. *G*(*q*, k) values ≥  0.75), except for Relevance Appraisal, where reliability was good (i.e. *G*(*q*, k) values between 0.6 and 0.74). As shown in Table 2, single-measure reliability was excellent for four domains: Research Obtained, Quality Appraisal, Generating New Research, and Imposed Use. Reliability was good for the remaining domains, except Relevance Appraisal, where reliability was fair (i.e. *G*(*q*, k) values between 0.4 and 0.59).

#### The reliability between the independent coders scores and the average of the experts

As shown in Table 2, the results revealed excellent single-measure reliability (i.e. *G*(*q*, k) values ≥ 0.75) for three domains: searching for research, quality appraisal, and imposed use. Reliability was good (i.e. *G*(*q*, k) values between 0.6 and 0.74) for four domains: Research Obtained, Generating New Research, Interacting with Researchers, and Tactical Research Use. For the remaining three domains—Relevance Appraisal, Conceptual Use, and Instrumental Use—reliability was fair (i.e. *G*(*q*, k) values between 0.4 and 0.59).

## Discussion

### Summary of findings

The results showed that experts could use the SAGE scoring tool to score the range of interviews/policy documents assigned to them. Most experts provided scores ranging from the limited to extensive bracket on the scale for all ten measured domains. The experts as a group also exhibited at least a fair level of agreement across all domains; however, for single-rater reliability, agreement was poor on three subscales: Relevance Appraisal, Conceptual Use, and Instrumental Use. Interrater agreement between the independent coders was good to excellent for all SAGE domains. Importantly, this was also the case for single-measure reliability, indicating that one of the coders could have accurately made reliable ratings on his/her own across all domains. Finally, interrater agreement between the independent coders and experts (averaged) was fair to excellent across all domains. These findings indicate a good level of agreement between the independent coders and the selected sample of experts as a whole, and that a single independent coder was scoring at a comparable standard to this expert sample as a group.

### Implications

Because of the small sample used in the present study, the implications made here are highly tentative. Nonetheless, the fact that experts as a group and individually provided scores that ranged from limited to extensive for all measured domains indicates that the SAGE scoring tool had sensitivity to discriminate between documents that were limited, moderate, and extensive in terms of research engagement and use. This provides evidence that the concepts being measured in SAGE were meaningful to a sample with expertise in the intersection between health policy and research, which suggests that the tool is comprehensible and possesses a good degree of content validity and acceptability. Demonstrating that the tool is comprehensible and usable among such experts is necessary if SAGE is to be used as a valid measure of research use in policy agencies.

The elevated level of agreement among the two independent coders points to the possibility that SAGE interviews can be scored with acceptable reliability and accuracy by a single rater without expertise in evidence-informed health policy. Another key finding was the fair to excellent agreement observed between the nine experts and two independent coders in this sample across all ten SAGE domains. This result implies that non-expert coders using SAGE may yield ratings that coincide with those made by health policy research experts (whose ratings we would consider to be the “gold standard”, or the best approximation to policymakers’ true engagement with and use of research in policy). The implication of this finding is that policy agencies wishing to use SAGE could train agency staff to score SAGE as opposed to recruiting expert coders, which would be both faster and more cost-effective for the agency. We emphasise, however, that further research with a larger sample of independent coders is required to confirm these implications.

There are few reliable and valid tools to measure research use in policy agencies, but are necessary for enabling agencies to evaluate and improve research use in policymaking. Considering the present findings, SAGE holds great promise for filling this gap. As a comprehensive measure of policymakers’ research engagement actions and use, SAGE can help shed light on the tools, systems, and structures agencies could invest in to improve the way in which staff engage with and use research in policy. Furthermore, SAGE could potentially be used to monitor and evaluate whether these tools and systems lead to significant improvements in the use of research in health policy formulation over time [16].

### Limitations

The first major limitation of the present study is the very small sample size. In order to make definitive conclusions that ratings made by independent coders align with those made by experts as a population (in other words, whether independent coders yield *valid* scores for policymakers’ research engagement actions and research use), future studies incorporating larger, randomly selected samples of health policy researchers are required.

A second limitation of the study was that *customised* transcripts were scored. This process of customisation may have produced biases in the content of the transcripts. For example, our coding scheme may have caused us to miss blocks of text relevant to research engagement actions and research use. We note, however, that we used detailed empirical definitions of terms as the basis of our coding scheme [23]. We also included as much of the original text that was even remotely relevant to each of the domains being scored. Nonetheless, we cannot exclude the possibility that the process of customisation biased the results in some way, especially given the absence of firm definitions of the key terms being coded (e.g. conceptual use, relevance appraisal).

Reliability overall was lower among the experts compared to the independent coders. This may have occurred because experts only rated a portion of the documents resulting in an ill-structured measurement design (ISMD) which produces lower reliability estimates [42]. Also, the experts had varying levels of experience and knowledge, which may have contributed to the greater disagreement in their ratings. Furthermore, experts may have experienced difficulties understanding the scoring rules as outlined in the Instruction Booklet. This is unlikely since none of the experts expressed such difficulties throughout the study or upon return of their completed ratings.

Reliability was particularly lower among experts for the Relevance Appraisal, Instrumental Use, and Conceptual Use domains. This is not unusual since there are no hard and fast guidelines as to how relevance of research should be appraised, or how best to use research conceptually when formulating policy. In addition, conceptual and instrumental use are overlapping, abstract concepts that have been defined inconsistently in the literature, and so are open to interpretation (e.g. [4, 30, 24–28]). Furthermore, although most previous measures focus on instrumental use, this is often assessed by vaguely asking policymakers to identify whether or not they use research to inform policymaking [4, 5, 7–9]. Because there is less clarity in the definition of these domains, the experts may have resorted to using their own idiosyncratic scoring rules, as was suggested by the results. These idiosyncrasies were most likely shaped by their specialised knowledge of different areas of health research and policy, possibly causing them to deviate from the prescribed scoring guidelines. (In contrast, because the independent coders were not experts in these areas, their ratings were most likely shaped by the scoring guidelines, thus contributing to greater consistencies between them). The idiosyncratic rules used by experts are not “noise” but meaningful variation that can be harnessed to produce a valid, reliable, and contextually sensitive scoring system and to guide best practices in measuring research use in health policy. Indeed, the SAGE scoring system was produced by systematically quantifying the opinions of over 50 health policy researchers using a choice experiment [13, 14]. However, given the breadth of health policies in vast fields of health, it is unlikely that SAGE has fully accounted for all the possible decision rules all experts use to score how research is used in health policy. Further systematic exploration of these decision rules and then using this information to produce versions of SAGE tailored for distinct health policy areas (e.g. mental health policy, public health policy) may produce a more extensive and broad-ranging system to score research use in health policy.

Because single-measure reliability was not as strong among the expert sample, a single expert might not be able to provide reliable and accurate scores, particularly for the three domains that showed poor reliability. We do not regard this as a problem because SAGE was not designed to be scored by health policy research experts, as this would not be feasible in an agency setting, particularly in relation to the cost and time required to recruit such experts. Experts were recruited in this study to provide a benchmark on which to compare ratings made by coders who are not experts in this field, thus providing preliminary indication that the SAGE tool could be used reliably by policymakers and other end users who are not research experts. We did find, however, that the reliability of experts’ *average* scores ranged from fair to excellent across all subscales. Therefore, if an agency decides that SAGE should be scored by experts, it would best be scored by more than one.

## Conclusion

The results showed firstly that experts in evidence-informed health policy research, both individually and as a group, could use the SAGE scoring tool to score interviews describing numerous types of policy documents. Furthermore, across all domains, their ratings encompassed the limited to extensive brackets of the rating scale. Secondly, ratings were fairly reliable in this expert group. Third, independent coders using the tool exhibited a high level of interrater agreement with each other and at least a good level of agreement with experts’ ratings on all domains. The study requires replication in a considerably larger sample of coders and experts but nonetheless indicate that SAGE could be used to provide agencies with an in-depth evaluation of their research engagement and use. The results of SAGE could be used as a basis for improving the agencies research capacity and use and, ultimately, the formulation of evidence-based policies and improvements in population health.

## Additional files

Additional file 1: SAGE Interview. (PDF 250 kb)

Additional file 2: Customised Transcript Template. (DOCX 21 kb)

Additional file 3: SAGE scoring checklist. (XLSX 42 kb)

Additional file 4: SAGE scoring instructions. (PDF 709 kb)

Additional file 5: Mean document scores and standard errors for all nine expert raters on each of the ten SAGE domains. (DOCX 162 kb)

Additional file 6: Histograms for the distribution of policy document scores on each of the ten SAGE domains. (DOCX 52 kb)

## Abbreviations

BIBD: Balanced incomplete block design
CIPHER: Centre for Informing Policy in Health with Evidence from Research
ISMD: Ill-structured measurement design
SAGE: Staff Assessment of Engagement with Evidence from Research
SPIRIT: Supporting Policy in Health with Research: an Intervention Trial
