# Supplementary material for: Preliminary testing of the reliability and feasibility of SAGE: a system to measure and score engagement with and use of research in health policies and programs
Source: Implement Sci. 2017 Dec 19;12:149. doi: 10.1186/s13012-017-0676-7 (PMC5735943; doi:10.1186/s13012-017-0676-7)
Supplement: Supplementary file 2 — Customised Transcript Template. (DOCX 21 kb) [file 13012_2017_676_MOESM2_ESM.docx]

Interview Summary Document

### DOCUMENT:

| **Section A: Research Engagement Actions** |
| --- |
| A.1 Searching for literature: Searching for or otherwise identifying research to inform policy |
|  |
|  |
|  |
|  |
|  |
|  |
| A.2 Accessing Literature (Types of Research Obtained): reflects the types of research and resources that are accessed |
|  |
|  |
|  |
|  |
|  |
|  |

| **Section A: Research Engagement Actions** |
| --- |
| A.3: Research Appraisal Actions – Appraising **Relevance:** Assessing whether recommendations, options, or interventions described in a piece of research, is applicable, compatible, or pertinent to the current policy issue and context/setting being considered |
|  |
|  |
|  |
|  |
|  |
|  |
| A.4: Research Appraisal Actions – Appraising **Quality:** Quality appraisal refers to the degree of confidence that can be placed on the conclusions drawn from studies and estimates of effects, and this is based on a number of key factors including its methodology, rigour, validity (i.e., statistical, construct, and internal), and credibility. Quality refers specifically to the standard of the evidence and the validity of the findings (i.e., a lack of bias) |
|  |
|  |
|  |
|  |
|  |
|  |

| **Section A: Research Engagement Actions** |
| --- |
| A.5: Generating New Research and Analyses: Plans or intentions to commission, collaborate in, or undertake new research or analyses to inform policy |
|  |
|  |
|  |
|  |
|  |
|  |

| **Section A: Research Engagement Actions** |
| --- |
| A.6: Interaction, collaboration, and communication with researchers through events, projects, networks, committees, etc. Health researchers are individuals, usually scientists, who conduct research in health (as distinguished from clinicians, experts, and health practitioners). |
|  |
|  |
|  |
|  |
|  |
|  |

| **Section B: Research Use** |
| --- |
| B.1: Conceptual Research Use: Use of research to provide new ideas, understanding or concepts that influence thinking about policy |
|  |
|  |
|  |
|  |
|  |
|  |
|  |

| **Section B: Research Use** |
| --- |
| B.2: Instrumental Research Use: Use of research to directly develop *content* (guidelines, strategies, recommendations, technical decisions, Initiatives, service delivery systems, models, etc.) or *direction* of policy |
|  |
|  |
|  |
|  |
|  |
|  |

| **Section B: Research Use** |
| --- |
| B.3: Tactical Research Use: Use of research to justify or lend weight to pre-existing preferences and actions. The research is being used to persuade others (e.g., key interest groups, key stakeholders, or the public), justify, or lend weight to an existing or pre-existing point of view/course of action/guideline/strategy, to give credibility to a policy document, or to place one’s own ideas on the policy agenda |
|  |
|  |
|  |
|  |
|  |
|  |

| **Section B: Research Use** |
| --- |
| B.4: Imposed Research Use: Use of research to meet organisational, legislative or funding requirements that research be used. |
|  |
|  |
|  |
|  |
|  |
|  |
