# Supplementary material for: Preliminary testing of the reliability and feasibility of SAGE: a system to measure and score engagement with and use of research in health policies and programs
Source: Implement Sci. 2017 Dec 19;12:149. doi: 10.1186/s13012-017-0676-7 (PMC5735943; doi:10.1186/s13012-017-0676-7)
Supplement: Supplementary file 4 — SAGE scoring instructions. (PDF 709 kb) [file 13012_2017_676_MOESM4_ESM.pdf]

# SAGE

Instructions on using the SAGE scoring tool to rate the extent of research engagement and use in the development of a policy document

## CONTENTS


Study background and instructions 4

- Background 4
- The Components of SAGE 4
- Purpose and Aims 6
- Instructions 8
- Additional Scoring Notes 10
- Confidentiality and Privacy 12

**Appendix** **13**

- Information about CIPHER 14
- Information about SPIRIT 16
- Information about SAGE 17
- SAGE interview questions 20
- Complete worked example 22

# STUDY BACKGROUND AND INSTRUCTIONS

## BACKGROUND

There are global calls to strengthen the capacity of organisations worldwide to transfer research evidence into healthcare practices and policies and several groups have developed tools and systems designed to facilitate this.

In order to evaluate efforts to increase the use of evidence in policy and program development, however, we require valid methods of measuring the extent to which evidence has been used. The Centre for Informing Policy in Health with Evidence from Research (CIPHER) (see Appendix p. 12) has developed an instrument known as SAGE (See Appendix p.18) to measure the extent to which policymakers engaged with and used research in the development of discrete policy products.

## COMPONENTS OF SAGE

SAGE consists of a ***semi-structured interview*** about a particular policy document, with the person (i.e., the policymaker) most heavily involved in developing the document (see Appendix p. 18). In the SAGE interview, the policymaker is invited to describe:

- The background to the document and the context in which it was developed
- Whether and how research was sought to inform the document
- The quality and relevance of the research that was found and how this was determined
- Whether plans were made to generate or advocate for generation of more research
- Whether and how research was used:
  - To help understand how to think about an issue (conceptually)
  - To make decisions about policy content or direction (instrumentally)
  - To persuade others to a point of view or course of action (tactically)
  - Because your organisation required you to use research (imposed)
  - In other ways?
- Barriers and facilitators to use of research in the development of the document.

Interviews are audio-recorded and transcribed. The interview transcript was then converted into a *customised transcript*, whereby responses were arranged into key sections: the six research engagement actions and four research use domains (see below).

The ***SAGE scoring tool*** (attached Excel File) allows objective assessors to rate, on the basis of policymakers' SAGE interview responses and the accompanying policy document, the extent of research engagement and use in the development of the policy document.

Specifically, SAGE requires coders to make ratings on the following six research engagement actions and four research use domains.

## RESEARCH ENGAGEMENT ACTIONS:

1. Searching for research: how would you rate the policy maker's strategy to search for research the development of the policy document?
2. Finding research: How would you rate the amount and type of research that the policy maker found when developing the policy document?
3. Appraising research – relevance:  how would you rate the policy maker's strategy of assessing the relevance of research to be used in the development of the policy document?
4. Appraising research – quality: how would you rate the policy maker's strategy of assessing the quality of research to be used in the development of the policy document?
5. Generating new research: how would you rate the policy maker's efforts to commission, collaborate in, or undertake new research or new analyses to inform the development of the policy document?
6. Interacting with researchers: how would you rate the policy maker's efforts to interact, collaborate, and communicate with researchers through events, projects, networks, committees, etc?

## RESEARCH USE:

7. Conceptual use of research: to what extent did the policy maker use research to provide new ideas, understanding or concepts that influence thinking about policy?
8. Instrumental use of research: to what extent did the policy maker use research to directly develop content or direction of policy?
9. Tactical/Symbolic use of research: to what extent did the policy maker use research to justify or lend weight to pre-existing preferences and actions?
10. Imposed use of research: to what extent did the policy maker use research to meet organisational, legislative or funding requirements that research be used?

## HOW THE INTERVIEW DATA YOU WILL BE SCORING WAS COLLECTED:

The data you will be scoring was collected as part of the SPIRIT trial (See Appendix p. 14). During each measurement point in SPIRIT agencies participating in SPIRIT were asked to provide four (4) documents which best demonstrated their use of research in development of a policy document in the preceding six months.

Semi-structured SAGE interviews were then conducted with the one or two policy makers who had the most substantial role in the development of each document. A copy of the interview schedule is provided in Document 5.

## PURPOSE AND AIMS OF THE STUDY

We have developed a system to score the SAGE interviews. The SAGE scoring system is like a checklist, such that if the policymaker undertook the specified action, it is ticked off in the checklist. We envision that scoring of SAGE data will ultimately be performed by one or two research officers. These research officers will be trained on how to use the scoring system to score policymakers' interviews and policy documents.

However, before a research officer can use the tool to score all the SAGE interviews, we need to establish whether the ratings made by the research officers, display a high level of inter-rater agreement with the ratings made by Experts in public health policy and research.

Thus, the aim of this study is to establish the inter-rater reliability of the SAGE scoring tool, which will also provide an indication of the tool's validity. Ensuring the measure is reliable and valid increases our confidence in the accuracy of ratings made by an independent assessor.

## PROCEDURE AND INSTRUCTIONS

1. You should have been sent the following materials
    a. This instruction booklet (PDF)
    b. 3 customised SAGE interview transcripts in PDF format
    c. The 3 accompanying policy documents/reference lists PDF
    d. SAGE scorecard (Excel file)
2. Please begin by reading through the SAGE scoring card Excel file to familiarise yourself with all the actions you will be rating. These key actions are highlighted in bold, with definitions and concrete examples provided to help you interpret each action. Reading through this scorecard will help you identify relevant actions undertaken by the policymaker when you read the interview transcripts in order to make your ratings.
3. About the materials:
    a. Each customised transcript has 10 domains divided into two main sections – Section A: Research Engagement Actions, which comprises of six domains: (1) searching for research, (2) types of research found and used, (3) appraising relevance, (4) appraising quality, (5) generating new research or analyses and (6) interacting with researchers; and Section B – Research use, comprising of four domains of research use: (7) instrumental, (8) conceptual, (9) tactical, and (10) imposed.
        i. The content within each section of the customised transcript was taken from many different sections of the original interview transcript, not just the questions that specifically addressed that particular domain
    b. The SAGE scorecard is also divided into these 6 Research Engagement Actions and 4 Research Use Domains.
    c. There is a separate scorecard for each document you will rate. These separate scorecards are located in individual sheets which can be accessed at the bottom of Excel (see Fig 1). Each sheet is labelled with the name of the specific policy document. Please make sure you score each interview/policy document in the correct sheet. Because you will be rating 3 documents, there will be 3 sheets in the Excel file (see Fig. A below)

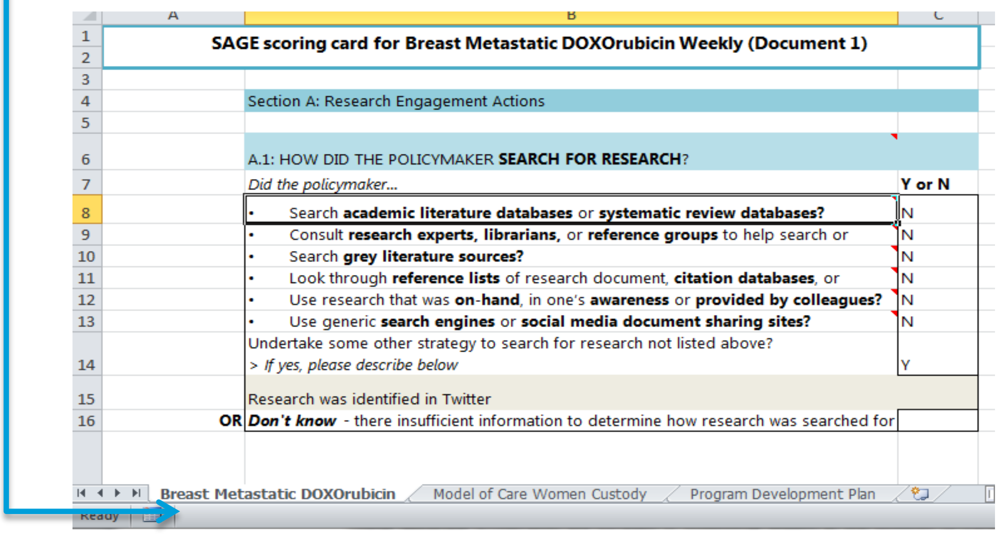


*Fig 1* Separate score cards for each document

d. The SAGE scorecard lists a series of actions that the policymaker may or may not have undertaken, for each research engagement action and type of research use. The actions are listed in a format similar to a checklist (see Fig. 1).

| | | |
|---|---|---|
| | A.1: HOW DID THE POLICYMAKER **SEARCH FOR RESEARCH**? | |
| | *Did the policymaker...* | **Y or N** |
| | • Search **academic literature databases** or **systematic review databases?** | |
| | • Consult **research experts, librarians,** or **reference groups** to help search or identify research? | |
| | • Search **grey literature sources?** | |
| | • Look through **reference lists** of research document, **citation databases**, or **reference manager databases (e.g., Endnote)?** | |
| | • Use research that was **on-hand**, in one's **awareness** or **provided by colleagues?** | |
| | • Use generic **search engines** or **social media document sharing sites?** | |
| | Undertake some other strategy to search for research not listed above? | |
| | *> If yes, please describe below* | |
| | | |
| OR | ***Don't know*** - there insufficient information to determine how research was searched for | |

*Fig 1.* SAGE scorecard for Section A.1 - Searching for research

## 4. Scoring

a. Start by opening the first transcript (labelled: Transcript 1: *document name)* and its accompanying policy document (in PDF format, with the same title). Proceed by reading through the transcript and rating it section-by-section in the SAGE scorecard.

b. For example, begin by reading the first section in the customised transcript, which is *searching for research*. You will then score this section using the SAGE scoring tool section "A.1: Searching for Research" (see Fig. 1)

c. For every action listed on the scorecard, if it is **clearly evident** from that interview transcript and/or the policy document that the policymaker undertook the action, select **Yes**, in the dropdown box next to that action. "Yes" indicates that *"yes, this action was performed by the policymaker"*.
**Remember:** Only rate "YES" if it is clearly evident that the action was undertaken.

If the policymaker did not perform the action listed in the scorecard, select **No** in the dropdown box beside that action. "No" indicates that *"No, this action was not performed by the policymaker"*.

For example in **Fig 2A** below: the policymaker has reported in his or her interview that he or she used academic databases such as PubMed, and also used Google. As a result, in **Fig 2B**, I have selected **Yes** in the dropdown boxes beside those two actions in the Excel scorecard: "searched academic literature databases or systematic review databases" and "used generic search engines or social media document sharing". For all those other actions that were not performed, I selected **No** in the dropdown boxes beside those actions

| | |
|---|---|
| Facilitator: | So where did you look? How did you go about that search? |
| Interviewee: | We either use some of the database that we've got access to ... |
| Facilitator: | Sorry I think I might have lost you for a minute or so there. |
| Interviewee: | Is that clear now? |
| Facilitator: | Yes. |
| Interviewee: | So usually Google's the first one, so trying to look for certain key words and what comes up. Then the databases that we have access to, that's what I would usually, my first point of call would be unclear PubMed. |

*Fig 2A.* Interview Transcript

| A.1: HOW DID THE POLICYMAKER **SEARCH FOR RESEARCH**? | |
|---|---|
| *Did the policymaker...* | **Y or N** |
| • Search **academic literature databases** or **systematic review databases?** | Yes |
| • Consult **research experts, librarians,** or **reference groups** to help search or identify research? | No |
| • Search **grey literature sources?** | No |
| • Look through **reference lists** of research document, **citation databases**, or **reference manager databases (e.g., Endnote)?** | No |
| • Use research that was **on-hand**, in one's **awareness** or **provided by colleagues?** | No |
| • Use generic **search engines** or **social media document sharing sites?** | Yes |
| Undertake some other strategy to search for research not listed above?<br>> *If yes, please describe below* | No |
| | |
| OR ***Don't know*** - there is insufficient information to determine how research was searched for | |

*Fig 2B.* SAGE scorecard

d. If the policymaker has undertaken an action that is not listed in the checklist, or you are having difficulty identifying if an action matches any of those in the checklist, select **Yes** in the dropdown box beside *"undertake some other strategy…"* and provide some details in the box below describing what exactly the policymaker did (See Fig 3).

| A.1: HOW DID THE POLICYMAKER **SEARCH FOR RESEARCH**? | |
|---|---|
| *Did the policymaker...* | **Y or N** |
| • Search **academic literature databases** or **systematic review databases?** | No |
| • Consult **research experts, librarians,** or **reference groups** to help search or identify research? | No |
| • Search **grey literature sources?** | No |
| • Look through **reference lists** of research document, **citation databases**, or **reference manager databases (e.g., Endnote)?** | No |
| • Use research that was **on-hand**, in one's **awareness** or **provided by colleagues?** | No |
| • Use generic **search engines** or **social media document sharing sites?** | No |
| Undertake some other strategy to search for research not listed above?<br>> *If yes, please describe below* | Yes |
| Research was located with a post on Twitter | |
| OR ***Don't know*** - there is insufficient information to determine how research was searched for | |

*Fig 3.* SAGE scorecard comments section

5. To help you decide whether or not a policymaker has performed the actions listed in the scoring guide, each action in bold is provided with a definition, and several concrete examples. These definitions are contained in comments boxes, which can be accessed by rolling over the relevant cell in the Excel scorecard (See Fig 4).
    a. For example, in Section A.1 searching for research, **academic literature databases** is in bold. Rolling over or clicking the cell will open a comments box containing definitions and examples of academic literature databases. Seeing as though PubMed is listed as one of the examples, this indicates the policymaker used Academic Literature databases to search for research

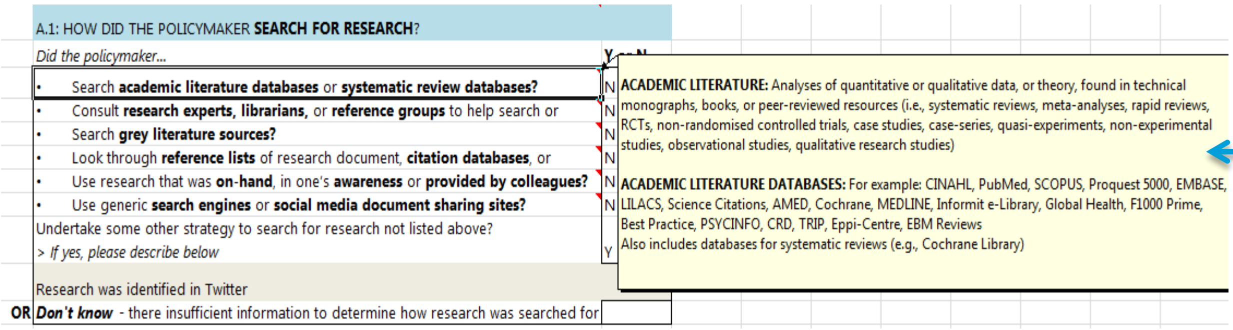


*Fig 4.* SAGE comments boxes

***Please read all the comments boxes before making your ratings***

**NOTE:** It may not always be possible to rate a particular section in the scorecard on the basis of the interview transcript. This is because, sometimes, the interviewee was not the primary author of the policy document, due to factors such as staff turnover or multiple authors contributed different components to the document. Consequently, the interviewee may not be able to provide all the required information to rate each dimension. In this situation, try your best to use the available information to make your rating.

When rating section A.2 Types of Research Found, please examine the policy document to see if there is a reference list or bibliography. This will help you determine the types of research that informed the development of the document. The reference list is useful for interviewees that are unable to describe the types of research that influenced the document's development. Please note, however, that many documents are not referenced even though research was used in their development.

6. Please repeat steps 3 and 4 for the remaining sections of the transcript/scorecard

7. If you have any questions, please feel free to contact me (ph: 0422 259 451 or by email: stevemakkar@saxinstitute.org.au)

8. Please send your Excel scorecard, with completed ratings for all 3 documents to steve.makkar@saxinstitute.org.au

## ADDITIONAL SCORING NOTES

- Only mark "YES" if it is ***clearly evident*** that an action was undertaken (unless stated otherwise in the comments boxes of particular items). If it is *likely* or *probable* that an action was undertaken but it was not explicitly stated, then mark "NO" for these actions (unless instructed otherwise in the scorecard and comments boxes). Please make a note of any uncertainties in the comments area below each section of the scorecard.
- ***Please do NOT extrapolate when making your ratings***. Only use the information within the transcripts to make your ratings. The SAGE scorecard and the comments (see step 5) provides details on how to rate when policymakers' comments are ambiguous for particular items
- ***Please read through the worked example (on page 23 onwards).*** This worked example highlights the relevant aspects of the transcript that correspond to the SAGE scorecard. We also provide screenshots of the SAGE scorecard, indicating the items that have been rated as YES and NO on the basis of the transcript.
- **Please do NOT leave any YES/NO boxes empty**

## ADDITIONAL SCORING NOTES FOR SECTION A: RESEARCH ENGAGEMENT ACTIONS

- Searching for Research and Accessing Research only refer to literature (grey or citable), and not any new research that was generated ***specifically*** for the purposes of the policy.
- Only give a score of 0 if a REA was not performed at all.
    - For example, in some instances, an action (e.g., searching for literature) was undertaken by consultants or another colleague. This does not deserve a score of 0 on searching for literature, because research was clearly undertaken. In this situation, please use the available information as best you can to make a rating.
- If insufficient information is available to make a rating on a particular dimension, please mark the "don't know" box on the SAGE rating sheet
- If research was not searched for at all, then you cannot make a rating on sections A.2-A.4
- If research was searched for, but then not used (section A.2), it is still possible to rate how they appraised the relevance and quality of the research.
- In numerous interviews, policymakers consulted or formed a steering group or working group consisting of "experts". Given that SAGE uses a broad definition of research findings (i.e., analyses of quantitative or qualitative data, or theory, found in peer reviewed papers, technical monographs or books, or in grey literature such as internal studies and evaluations, and reports on authoritative websites), we will consider a broad definition of researcher to include **experts** and **clinicians/practitioners/doctors** (this does NOT include managers or senior managers).
    - These individuals would most likely be up-to-date with the relevant research in the area.

- The interviewee must **explicitly mention** "experts" or "clinicians" or "practitioners" or "doctors"

## ADDITIONAL SCORING NOTES FOR SECTION B: RESEARCH USE

- Research includes the types of research identified in section A.2 (Types of Research Found) as well as **formally generated research** (i.e., rapid reviews, formal studies and investigations including those described in Section A.5 – *Thorough Research Generation Activities*, and section A.6 – *Thorough Collaborative Activities with Researchers*).
  - Therefore, **this does not include** non-formalised meetings, stakeholder consultations, advisory group meetings, meeting minutes, or other *informal research generation activities.*
- If research was neither searched for, accessed, nor generated, then score 0 on all dimensions of Research Use

## GENERAL THINGS TO BE AWARE OF

Please be mindful that:

- The agencies participating in SPIRIT undertake a diverse range of policy and program work in widely varying settings.
- The agencies were asked to select policy documents that best demonstrated their use of research in policy development.
- Agencies use many different sources of information to develop their policy and program documents, of which research is only one. The role and importance of research also varies with the nature of the policy document being developed.
- The use of research may not be overtly displayed in the final policy document since, for example, there may not be references to research in the document. However, we are interested in rating the use of research *during the development of the document* rather than in the extent to which this is visible in the final document. So, if you judge that research was used in the development of the document then you should rate it accordingly even if the research is not explicitly mentioned in the document
- The research team have asked for the best evidence available and probed for details wherever possible. It is not possible to collect more evidence from the agencies.  Assessments must be drawn on the documents and interview summaries provided.

## CONFIDENTIALITY AND PRIVACY

Participants are reminded of the importance of confidentiality and privacy when rating the transcripts and policy documents of agencies.  Ethical approval for this study was granted with the condition that the identity of panel members is provided to the UWS ethics secretariat and that all information disclosed to the Panel would remain confidential.  Results will be reported with agencies and individuals de-identified. We require that participants do not disclose to any person the performance of each agency outside the secretariat and CIA of the study (Sally Redman). All the information contained in the policy documents and interviews is to be treated as strictly confidential.

# APPENDIX

## WHAT IS CIPHER?

### CIPHER IS:

- An NHMRC funded Centre of Research Excellence - the Centre for Informing Policy in Health with Evidence from Research
- Funded for five years (until April 2016) with a total amount of $2.5M
- A collaboration between research organisations including many leading researchers

### CIPHER HAS BEEN ESTABLISHED BECAUSE:

- It is widely accepted that research can make a significant contribution to policy development and evaluation
- Currently many opportunities to consider research findings in policy development are missed
- There is very little evidence about how to increase the capacity of policy agencies to use research as effectively as possible

### CIPHER WILL:

- Develop and test the impact of new, intensive strategies to increasing the effective use of research in policy
- Develop and test methods of measuring and describing research utilisation in policy
- Work closely with interested policy agencies to develop tools, skills and systems that will increase their capacity to use research effectively in their policy work
- Provide feedback to participating agencies about their use of research in policy making
- Stimulate discussion about how best to increase the effective use of research in policy
- Develop and test new approaches to increasing researchers' capacity to:
    - Produce research that is useful in policy decision-making
    - Generate accessible summaries of research findings
    - Work effectively with policy agencies

### CHIEF INVESTIGATORS:

**Professor Sally Redman, Sax Institute, (CIA)** brings expertise in approaches to increasing the effective use of research in policy and in evaluation design.

**Professor Louisa Jorm, Foundation Professor of Population Health, University of Western Sydney & Principal Scientist, Sax Institute,** brings extensive expertise in policy-relevant research and in working with policy agencies to broker research and research partnerships.

**Professor Sally Green, Co-Director of the Australasian Cochrane Centre and a Professorial Fellow at Monash University,** is an established leader in increasing the effective use of research in clinical practice and policy.

**Professor Cate D'Este, Chair of Biostatistics, University of Newcastle,** has extensive experience in working with policy agencies and in the statistical methods required for analysis of complex interventions.

**Ms Deborah Frew, NSW Department of Health,** is very experienced in government policy making and in facilitating the effective use of research in health policy.

**Professor Anthony Shakeshaft, Assistant Director, National Drug and Alcohol Research Centre, University of NSW,** has experience of implementing and evaluating practical intervention strategies in a wide range of settings, and of working closely with policy agencies.

**Professor Rob Sanson-Fisher, Director Priority Research Centre for Health Behaviour, University of Newcastle,** brings internationally leading expertise in approaches for health behaviour change, and development, implementation and evaluation of health service models.

**Professor Huw Davies, Professor of Health Care Policy and Management, University of St Andrews, Scotland,** is an international leader in examining the role of research evidence in health care policy, service design and professional practice.

**Professor Jordan Louviere, Professor of Marketing and Executive Director, Centre for the Study of Choice, University of Technology Sydney,** brings extensive expertise in the use of choice methodologies for evaluating measures and potential new strategies.

## ASSOCIATE INVESTIGATORS:

**Professor Alex Barrett, School of Public Health, University of Sydney,** is a leading epidemiologist with expertise in the communication of evidence from health research for decision making.

**Dr Terry Flynn, Senior Research Fellow, Centre for the Study of Choice at the University of Technology Sydney** is a senior researcher addressing methodological and empirical issues in choice modelling.

**Associate Professor Mary Haines, Senior Research Fellow, Sax Institute** is a leading health services researcher with expertise in implementation research.

**Dr Denise O'Connor, Senior Research Fellow, NHMRC Public Health Fellow at the Institute of Health Services Research at Monash University,** has expertise in the measurement and change of health behaviour to increase evidence uptake and is an editor for the Cochrane EPOC group.

**Ms Sarah Thackway, Director Epidemiology and Research, NSW Department of Health,** brings extensive expertise in policy development and the integration of research and policy.

**Associate Professor Fiona Blyth, Coordinator of Medical Education, Concord Clinical School, University of Sydney**

**Dr Andrew Milat, Associate Director, Centre for Evidence and Evaluation, NSW Ministry of Health.**

**Dr Stacy Carter, Centre for Values, Ethics & Law in Medicine (VELIM), University of Sydney.**

spirit Supporting Policy In health with Research: an Intervention Trial

## INFORMATION ABOUT SPIRIT

## SPIRIT: SUPPORTING POLICY IN HEALTH WITH RESEARCH: AN INTERVENTION TRIAL

### WHAT IS SPIRIT?

SPIRIT is an NHMRC-funded trial of the impact of our 'best bet' strategies to help agencies use evidence from research in their work. It has been established because there is currently no information about what strategies may be most useful to agencies interested in increasing the use of research evidence.

### WE EXPECT THAT SPIRIT WILL RESULT IN:

- Findings of international interest about what works to increase the use of research in policy.
- Advice to our network of policy agencies in NSW about how they might best consider changing their practice, programs or policies.
- A valuable opportunity for participating agencies to find out about their current practice and to access free of charge leadership and training programs, exchanges with researchers, and review, and analysis services

### SPIRIT IS AN INITIATIVE OF...

The *Centre for Informing Policy in Health with Evidence from Research (CIPHER)*, a Centre of Research Excellence funded by the NHMRC. CIPHER is a collaboration between nine Australian and international universities. CIPHER is working with a network of policy agencies across NSW to explore and share information about how best to increase the effective use of research in policy.

### SPIRIT IS BEING IMPLEMENTED...

In six policy agencies in NSW. It commenced in October 2012. We are using a 'step wedge' design, in which each of the six agencies:

- Receives over 12 months a 'Research for Policy Program'. The Program includes a mix of activities and services aimed at facilitating better use of research in policymaking.
- Will be randomly selected to commence the Research for Policy Program at one of three start points across the trial period.
- Will participate in outcome measurement at six time points across the trial period, including before, during, and immediately after the Research for Policy Program, and during a sustainability follow-up period.
- Will have its research use compared over time against its own previous measurements. There will be no traditional 'control' agencies which do not receive the Program; rather, each agency will function as its own control

## INFORMATION ABOUT SAGE

SAGE is an interview about the development of a policy/program-related document. Following is the information sheet provided to participants who completed the SAGE interviews.

## WHAT?

### WHAT IS THE SAGE INTERVIEW ABOUT?

SAGE is an interview about how research was or wasn't used in the development of a policy or program document (i.e. a document that was written as part of a policy or program development process). It asks about how research was sought, appraised and applied, and seeks to understand the contextual factors that may have affected this process. We are using SAGE as one of the ways of measuring the use of research in policy and program development. We will summarise findings from SAGE and provide feedback to your organisation.

## WHY

### HOW DOES SAGE RELATE TO SPIRIT?

SAGE is one of the measurement tools being used in the SPIRIT study. The purpose of SPIRIT is to see if a program of capacity-building activities and resources is able to support agencies to use research more effectively in health policy and program development. SPIRIT comprises the Research for Policy program (R4P), three outcome measurement tools (SEER, ORACLe and SAGE), use of a knowledge exchange portal (Web CIPHER), plus a process evaluation and cost analysis.

How was I selected to participate in a SAGE interview?

Policy/program documents were identified by the person in your agency who is acting as the liaison person for SPIRIT. The liaison person selected four documents that your agency has produced in the last six months which they considered to be the best examples of research-informed policy or program development. Your liaison person has identified you as one of the policy makers or program developers who played the most substantial role in the development of one or more of these documents and they have provided us with your contact details so that we can ask you if you would be willing to be interviewed.

## HOW?

### WHAT DOES PARTICIPATION INVOLVE?

Four staff members will each be required to participate in one SAGE interview about the use of research in developing a specific policy/program document, at each of the six measurement points (every six months). The SAGE interview will take about 45 minutes and, with your permission, it will be audio recorded. The interview can be completed by telephone or face-to-face. If you are willing to complete an interview, we will arrange a time that is convenient to you.

## WHO/WHAT IS ELIGIBLE?

### SAGE DOCUMENTS

The four nominated documents should best represent the agency's capacity to use research in policy or program development. To be eligible a document must:

- be a review, report, discussion paper, draft or final policy, formal directive, program plan, strategic plan, ministerial brief, budget bid, service agreement, implementation plan, guideline or protocol *and*
- focus on health service or program design, delivery, evaluation or resourcing *and*
- have been produced in the last 6 months
- does not have to relate to a completed policy or program, but it does have to be finalised and signed off by its final signatory

## SAGE INTERVIEWEES

We want to talk to the person who had the most substantial role in developing the document. If two people contributed equally, both may be nominated.

## WILL THE STUDY BENEFIT PARTICIPANTS?

The SAGE interview is just one part of SPIRIT. As a participant in SPIRIT you will be given access to a range of agency-specific capacity development activities, services and resources within the Research for Policy program (R4P). You will also be given access to the Web CIPHER knowledge exchange portal.

SPIRIT will also benefit your agency which will receive a summary of how it currently uses research and free-of-charge participation in R4P at an estimated value of $70,000.

## WILL THE STUDY INVOLVE ANY RISKS?

No. We do not anticipate any risks or discomfort for you.

## HOW WILL THE INFORMATION BE USED?

Information gained during the study will be fed back to participating agencies, but this will be in an aggregated de-identified form that protects the identity of individual participants. Summaries of the results may be published in journal articles or conference papers reporting on the development of the tools and strategies, but neither individuals nor their agency will be identifiable.

Only the researchers will have access to information on participants. The data will be stored securely for 7 years after completion of the project and then disposed of.

## HOW DO I GIVE CONSENT TO PARTICIPATE?

When we send the invitation for SAGE we ask that you reply to the email saying that you consent or do not consent to participate in a SAGE interview. If you consent we will contact you to arrange a convenient time. If you do not consent we will not contact you again about SAGE.

## WHO IS RUNNING SPIRIT?

SPIRIT is being coordinated by the Centre for Informing Policy in Health with Evidence from Research (CIPHER), a Centre of Research Excellence funded by the National Health and Medical Research Council. CIPHER is a collaboration of nine Australian and international universities and policy makers led by Professor Sally Redman. The University of Western Sydney is the administering institution.

CIPHER is funded through a grant from the Australian National Health and Medical

Research Council from 15 October 2010, application number APP1001436.

## CAN I WITHDRAW FROM THE STUDY?

Participation is entirely voluntary: you are not obliged to be involved and, if you do participate, you can withdraw at any time without giving any reason and without any consequences.

## CAN I TELL OTHER PEOPLE ABOUT THE STUDY?

Yes, you are free to tell other people about the study. You and they are welcome to contact the chief investigator to discuss the research project.

## WHAT IF I REQUIRE FURTHER INFORMATION?

If you would like to know more at any stage, please contact the SPIRIT Project Officer at the Sax Institute on (02) 9514 9243 or SPIRIT@saxinstitute.org.au.

## WHAT IF I HAVE A COMPLAINT?

This study has been approved by the University of Western Sydney Human Research Ethics Committee. The approval number is H9870. If you have any complaints or reservations about the ethical conduct of this research, you may contact the Ethics Committee through the Office of Research Services on Tel +61 2 4736 0229 Fax +61 2 4736 0013 or email humanethics@uws.edu.au. Any issues you raise will be treated in confidence and investigated fully, and you will be informed of the outcome

## SAGE INTERVIEW QUESTIONS – LIST VERSION

1. Can you tell me about the document that we are going to discuss today?
2. Can you explain for me why this document was selected for a SAGE interview?
3. What was your role in the development of this document?
4. Did you, or others, look for research to inform development of this document?
5. Can you describe how you or others looked for research to inform this document? How was research sought/ found/accessed/identified? What was actually done to get research? Where did you look?
6. Was any research found?
7. What types of research were found?
8. Thinking first about the relevance of the research to this document, how relevant was the research that was found?
9. How did you, or others, work out whether the research was relevant or not? How was the relevance of the research determined? Were there any specific criteria for working out whether research was relevant? Was there a standard process for working out whether research was relevant?
10. Thinking now about the quality or reliability of the research, what was the quality of the research that was found?
11. How did you, or others, evaluate the quality of the research? Were there any specific criteria for assessing the quality? Was there a standard process for assessing the quality? How did you determine whether the research was reliable or robust?
12. Did you, or others, make plans to commission, conduct or advocate for more research as part of development of this document?
13. Did research inform the development of this document in any way?
14. How did you, or others, use research in the development of the policy document? We are interested in the different ways research can be used in policy/program development, for example, was research used to [...Insert sub-questions here...]? Can you describe how you used research in that way?
15. Were there any barriers to using research in the development of this document? What were they? What impact did they have? How much of a problem were they?
16. Were there any things that helped you use research (facilitators/enablers) in the development of this document? What were they? What impact did they have? How much help were they?

17. Could research have been more helpful in the development of this document?

18. Do you have any concerns about how research was or wasn't used in the development of this document?

19. On a scale of zero to five; with zero being "Played no role" and five being "Essential", how would you rate the importance of research in the development of this document?

20. How representative is this document of policy/program development in your organisation (particularly in relation to the use of research)?

21. Do you have any other comments about the use of research in the development of this document?

22. Do you think the results of this evaluation will impact on future program or policy development

Interview Summary Document – Worked example

In this worked example, we have highlighted relevant quotations in the transcript that address each of the constructs measured in SAGE. We have added comments to show how these quotations map onto the different actions measured in the SAGE scorecard. We have also provided screenshots of the scorecard for each section showing the items we ticked off based on the relevant quotations that were highlighted.

DOCUMENT: **eviQ Patient Information Quality Improvement Project Proposal.** This document is proposal for a project to review the information available for patients on the eviQ website; This document includes an evaluation plan, to evaluate and improve the patient information on eviQ.

| Section A: Research Engagement Actions | |
|---|---|
| A.1: Accessing Research | |
| i) Searching for literature: Searching for or otherwise identifying research to inform policy | |
| Facilitator: | Could you describe the details of the methods that were used to search for research for this document? |
| Interviewee: | So I did the kind of **usual literature search looking through the databases so PubMed, CINAHL, Embase**, looking through all of those, looking for stuff that was - the literature that was relevant to patient information and quality review as well. **I was aware of the two documents that have already reviewed eviQ**. So I was aware of those and it was just through sort of wider searching that I was able to find things that I thought were appropriate to bring in to inform the development of the document. |
| Facilitator: | Yes. So did you have a plan for how you would search for the research or... |
| Interviewee: | Not really. I mean I was in the middle of doing my Masters at the time so I just used the same sort of - I mean I don't think I had a formulated plan but I had a message which I probably couldn't articulate. But I just had a way of actually doing things and then if I - but I would put in key words and then I would search on those key words. Then if I found things that were interesting from those key words or articles that were interesting from that then **I'd use those articles to have a look at what their references were.** See if they were - **so I think that hand searching and it's looking through the documents I found important or I felt very relevant to see if there was anything more relevant that I could use within those.** |
| Facilitator: | Yes. **You searched in the government databases, your own, you said about that like internal evaluations**. |
| Interviewee: | Yeah. I also **searched through government documentation as well in Australia and overseas.** |
| Facilitator: | Just the last one on my list is did you search for any books or small focus documents such as technical monographs? |
| Interviewee: | Well there's the [Agency] consumer engagement framework which I looked through. I **also went to some of the support websites, as in the international support websites so the NCCN and Macmillan in the UK**. They have some information around producing patient information so I referred to that. So yeah those were the other kinds of documents that I used to inform the background rather than actually used to produce a document. |

**Comment [SM1]:** She has searched **academic literature databases**

**Comment [SM2]:** Used **on hand research** as a starting point

**Comment [SM3]:** **Looked through references of articles** that he or she had found

**Comment [SM4]:** **Searched through grey literature sources** (government databases)

**Comment [SM5]:** This is an internal document, we **assume that it was obtained on hand** (based on the scoring guidelines)

**Comment [SM6]:** Looked through **grey literature sources – authoritative websites**

| Section A: Research Engagement Actions | |
|---|---|
| A.1: HOW DID THE POLICYMAKER **SEARCH FOR RESEARCH**? | |
| *Did the policymaker...* | |
| • Search **academic literature databases** or **systematic review databases** or **physical libraries?** | Yes |
| • Consult **research experts, librarians,** or **reference groups** to help search or identify research? | No |
| • Search **grey literature sources** (local intranet, authoritative websites, agency websites, grey literature databases or trials)? | Yes |
| • Look through **reference lists** of research document, **citation databases**, or **reference manager databases (e.g., Endnote)?** | Yes |
| • Use research that was **on-hand** or in **his/her computer/bookshelf/desk**, in one's **awareness**, through **email alerts, conferences**, or **provided by colleagues?** | Yes |
| • Use generic **search engines** or **social media document sharing sites?** | No |
| Search for research in locations not listed above? Please type them here --> | |

| ii) | Accessing Literature: reflects the types of research and resources that are accessed |
|---|---|
| Interviewee: | Yeah. **I also searched through government documentation as well in Australia** and overseas. |
| Facilitator: | Okay. What did you find there? |
| Interviewee: | I found **that there were a few documents pertaining to the involvement of co-consumers in research and the development of consumer related information. I found there were some guidelines.** In the UK there's some nice guidelines around reviewing documents. It's a while ago now so I'm scraping my memory banks [laughs]. |
| Facilitator: | Yes. You searched in the government databases, your own, you said about that like internal evaluations. |
| Interviewee: | Yeah. I also **searched through government documentation as well in Australia and overseas.** |
| Facilitator: | Just the last one on my list is did you search for any books or small focus documents such as technical monographs? |
| Interviewee: | **Well there's the [Agency] consumer engagement framework which I looked** through. I **also went to some of the support websites, as in the international support websites so the NCCN and Macmillan in the UK**. They have **some information around producing patient information** so I referred to that. So yeah those were the other kinds of documents that I used to inform the background rather than actually used to produce a document. |
| Interviewee: | So I did the kind of usual literature search looking through the databases so PubMed, CINAHL, Embase, looking through all of those, looking for stuff that was - the literature that was relevant to patient information and quality review as well. **I was aware of the two documents that have already reviewed eviQ. So I was aware of those and it was just through sort of wider searching that I was able to find things that I thought were appropriate to bring in to inform the development of the document.** |
| Facilitator: | Were you looking for primary research studies like trials or were you looking for reviews? What sorts of things? |
| Interviewee: | **Well it was both really.** I mean I think the thing is with this kind of area it's more of a qualitative area so it's not so much there was a primary research that we would use when we're developing protocols for eviQ. So I think in that respect it's slightly different. |

**Comment [SM7]:** Because there is no reference list, we **assume the documents were up-to-date**

**Comment [SM8]:** Used **grey literature external** to the organisation (govt documentation)

**Comment [SM9]:** **Grey literature obtained from external organisations** in the UK (i.e., guidelines)

**Comment [SM10]:** **External grey literature** (the government)

**Comment [SM11]:** **Internal policies** used

**Comment [SM12]:** Again, **grey literature obtained from external orgs**

**Comment [SM13]:** EviQ is part of this agency, **so this is an internal grey literature** (policy) document

**Comment [SM14]:** This indicates that he or she was looking for primary research (both quantitative and qualitative) and reviews, but didn't appear to use them.

| A.2: WHAT **TYPES OF RESEARCH** DID THE POLICYMAKER FIND AND USE? | |
|---|---|
| *Did the policymaker find and use...* | |
| • **Systematic reviews** and/or **meta-analyses?***** | No |
| • **Published primary research, qualitative (non-systematic) reviews** and/or **theoretical articles?** | No |
| • **Grey literature from <u>external</u> organisations:** Policies, evaluations, guidelines, research findings (published/unpublished), reports, data? [Includes **data from registries**] | Yes |
| • **Unpublished research studies** (e.g., dissertations, theses, abstracts) obtained from databases that specialise in unpublished research (e.g., OpenGREY, Open SIGLE database, Dissertation Abstracts International), or **conference resources?** | No |
| • **Books** and/or **technical monographs?** | No |
| • **Grey literature produced <u>internal</u> to the organisation:** Policies, evaluations, research (published/unpublished), reports, or data? | Yes |
| • **Recent (up-to-date)** as opposed to **dated** research from the above categories (that is, at least 50% of resources collected)? | Yes |
| Obtain resources that do not fit into the categories above. Please type them here --> | |

| Section A: Research Engagement Actions | |
|---|---|
| A.2.1: Research Appraisal Actions – Appraising **Relevance:** Assessing whether recommendations, options, or interventions described in a piece of research, is applicable, compatible, or pertinent to the current policy issue and context/setting being considered | |
| Facilitator: | Yes, I understand that. So now I'd like to ask you about whether you assessed the relevance of the research that you found and if so how did you do it? What aspects did you look at to determine how relevant the research was? |
| Interviewee: | **Well I looked at whether it was related to patient information and whether it was related to cancer patients. The type of patients that were involved in the research or involved in the reviews as well, the number and whether I felt that they were relevant to the Australian context as well.** |
| Facilitator: | That's good. Did you use any specific criteria or rating scales to determine how relevant the research was? |
| Interviewee: | **I have a kind of a - I wouldn't say a working knowledge, that's a little bit advanced but the CONSORT. It's CONSORT isn't it for the appraising literature research?** |
| *[note that CONSORT is a tool to assess the quality of reporting of RCTs and is NOT a relevance appraisal tool]* | |
| Facilitator: | Yes. |
| Interviewee: | So I used kind of that kind of framework to how I looked at the research to whether I thought that it was relevant or not and whether it answered the questions that it actually asked. So that sort of thing if that's what you mean. |
| Facilitator: | Yes, that's good. So overall how relevant did you find the research? |
| Interviewee: | There was a lot of research. A lot of research, let me assure you and it took much time and wading through and several days of kind of just looking at the stuff that I got back to determine whether it was relevant or not. **I really did have to whittle it down to just a few kind of reasonably relevant documents for my purpose.** When you're putting something like patient information I'm sure you can appreciate that's a huge, huge topic so you get tens of thousands of things come back and it's finding the stuff that's relevant. Because you don't want to sift through 20,000 documents, read all - I mean it's an impossible task to read all of them in that time period. But it's just a question of - **it was really the ones that stood out that I thought yeah that seems like it's relevant.** Does that answer your question? Sorry I went off on a tangent. |
| Facilitator: | So you may or may not have done this but did you document how you assessed the relevance of the research and what you found? |
| Interviewee: | No, I **didn't document it.** |

**Comment [SM15]:** He or she is looking at **aspects of the policy context** – such as the relevance to the Australian setting and the target population

**Comment [SM16]:** This is a tool for assessing quality. As a result, I am not rating this as an action to assess relevance

**Comment [SM17]:** Looked for resources to suit his or her purposes, in other words, research that **addressed the particular policy issue or needs.**

**Comment [SM18]:** This indicates that his or her approach was **not necessarily a preplanned, pre-specified, or explicit approach.**

**Comment [SM19]:** This shows that he or **she didn't document their approach**, further indicating that it was not a pre-specified approach to appraise relevance.

| A.3: HOW DID THE POLICYMAKER **APPRAISE THE RELEVANCE** OF THE RESEARCH? | |
|---|---|
| *Did the policymaker...* | |
| • Assess whether the research was **applicable to the policy context, policy priorities, needs, or policy issue/question/direction,** and/or was **timely/recently published**?** | Yes |
| • Use a systematic and **structured relevance appraisal guide?** | No |
| • Assess whether research recommendations were **actionable** and/or **feasible?** | No |
| • Consult **experts** to assess the relevance of research? | No |
| • Assess whether the research was **consistent with previous or current research findings** on the issue? | No |
| • Assess if research was compatible/consistent with his/her OR the organisation's **values, knowledge, actions, or experience?** | No |
| • Clearly undertake these actions as part of a **pre-specified** and **documented strategy** | No |
| Use strategies to appraise relevance that are not listed above? Please type them here --> | |

| A.2.2: Research Appraisal Actions – Appraising **Quality:** | |
|---|---|
| Facilitator: | That's good. Did you use any specific criteria or rating scales to determine how relevant the research was? |
| Interviewee: | **I have a kind of a - I wouldn't say a working knowledge, that's a little bit advanced but the CONSORT. It's CONSORT isn't it for the appraising literature research?** |
| *[note that CONSORT is a tool to assess the quality of reporting of RCTs and is NOT a relevance appraisal tool]* | |
| Facilitator: | Yes. |
| Interviewee: | So I used kind of that kind of framework to how I looked at the research to whether I thought that it was relevant or not and whether it answered the questions that it actually asked. So that sort of thing if that's what you mean. |
| Facilitator: | I can imagine. Now I'd like to ask about whether you assessed the quality and reliability of the research that you found and if so how did you do this? So what... |
| Interviewee: | So similar kind of methods as I've already described really. I don't think I did anything specifically, it was just whether... **did the research meet the aims of what the question was, that sort of thing... whether the questions asked were relevant to answer that question.** |
| Facilitator: | So aspects of quality sometimes people look at the type of study and... |
| Interviewee: | Yeah. |
| Facilitator: | ...how well it was conducted and the measures. |
| Interviewee: | Yeah. |
| Facilitator: | **Did you go into that much detail or...** |
| **Interviewee:** | **I didn't really drill down but if I looked at something and I thought that just doesn't look right, the methodological rigour isn't there and that sort of thing then I would just be - that would be my filter for discarding it.** So I only looked at the stuff that I thought was relevant and of good quality. |
| Facilitator: | Yes. **Did you use any specific criteria or rating scales to determine the quality of the research?** |
| **Interviewee:** | **Really again, it was going back to the CONSORT stuff as well.** That was really what I used as my guide because that's what I was used to using. |
| Facilitator: | Yes, that's good. So overall what was the quality of the research that you found? |
| Interviewee: | As I say I found a lot of research and the quality was a big range so there was a big range of quality. But I think the stuff that I used was of a good quality and helped me to inform what my purpose is for the project that I was undertaking. |
| Facilitator: | Yep, that makes sense. So you may or may not have done this but did you document how you assessed the quality and reliability of the research? |
| Interviewee: | No. |

**Comment [SM20]:** This shows that he or she used a **systematic and structured quality appraisal guide (CONSORT)** to appraise quality of the research.

**Comment [SM21]:** The person is **appraising quality by evaluating whether the nature of the research question –** whether it was appropriate to address the particular aims

**Comment [SM22]:** **Claims to have examined aspects of the study design** – in this case the methodological rigour

**Comment [SM23]:** Again, showing that **he or she used CONSORT – a structured tool to assess research quality.**

The use of a structured guide implies that their **approach was pre-specified and documented.**

| A.4: HOW DID THE POLICYMAKER **APPRAISE THE QUALITY** OF THE RESEARCH? | |
|---|---|
| *Did the policymaker...* | |
| • **Examine** aspects of the **study design** (e.g., methodology, statistics, sample selection) OR **assess** whether the aspects of the **study design were valid** OR whether **conclusions** of the research were **valid**? | Yes |
| • Use a **systematic** and **structured quality appraisal guide**? | Yes |
| • Assess the **level of evidence** of the research? | No |
| • Consult **experts** to assess quality or determine whether the **research was regarded as high quality** by **experts** in the field? | No |
| • Evaluate whether the **research question, design** or **conclusions** of the research were **described clearly** and **comprehensively,** and/or **were sufficient to address the purpose/aims** of the study? | Yes |
| • Assess whether the **source of the research** (the researchers, the institution) **was credible,** and/or if the research was **peer-reviewed,** or had undergone a **thorough and stringent process of review?** | No |
| • Check if the research:<br>- **cited/referenced** other high-quality research or policy documents;<br>- **was referenced in** other high-quality research or policy documents;<br>- came to **comparable conclusions** to other high quality research; OR<br>- was **reliable**? | No |
| • Clearly undertake these actions as part of a **pre-specified** and **documented strategy** | Yes |
| Use strategies to appraise quality that are not listed above? Please type them here --> | |

Section A: Research Engagement Actions

A.3: Generating New Research and Analyses: Plans or intentions to commission, collaborate in, or undertake new research or analyses to inform policy

Facilitator: Well given the amount of research that you've found or interesting findings did you think oh well let's plan to advocate or conduct more research?

Interviewee: Yeah I mean it's interesting that you ask that and I haven't - in my head is the kind of - because what we've done is we've done this as a quality improvement measure at one site. But having been to several conferences and people are aware of the project because it's one site for patients but it's on the website for clinicians. **I've had quite a lot of comments from clinicians saying "we would love to get our patients to do this". So yeah, possibly in the future but it's just the amount of time that it takes and looking at the logistics of it because I'm doing this on top of my usual work.**

Facilitator: Yes, you're very busy.

Interviewee: Yeah. Like everybody else do, you do your business as usual but this is a kind of extra stuff on the top as well. So I need to have some critical discussions, shall we say, regarding how [unclear] - I'd be very keen to but just whether we could - well it's just around the logistics of...

Facilitator: Whether it's possible.

Interviewee: ...if we do, do it how we do it and making sure that we do it in a way that's going to be meaningful, relevant and rigorous so those are some things I've got to consider. Because actually undertaking it now it's kind of like there were a couple of things that I probably would do differently if I was doing it again. But it's like anything isn't it, you don't really know until you actually try it

[doesn't mention any specific research activities, and indicates an uncertain intention]

Well I think to gain an understanding of how people have approached patient information. Its development and its review and evaluation previously so that informed some of the background but a lot of the kind of methodological stuff. I thought originally I would just send out a paper questionnaire but then I - **well I'm probably going to get more meaningful information if we do actually have a chat with people** because you can explain what you mean by your question if they're not grasping it. That was something that came through in the research and in discussion with the principle investigator as well. **They thought it would be more worthwhile if we did actually do it via a telephone interview for the patient portion. Obviously the clinician portion is just via a survey on the website**

**Comment [SM24]:** The interviewee mentions conducting **a thorough research generation activity (to conduct the evaluation on a larger scale) –** however, indicates that these **plans are uncertain.**

**Comment [SM25]:** Here she is providing more details about the intended research and the methods, further indicating that there is a possibility of **conducting a thorough research project.**

| | | |
|---|---|---|
| | A.5 WAS **NEW RESEARCH OR DATA ANALYSES GENERATED** TO INFORM THIS POLICY? | |
| | *Did the policymaker...* | |
| | • Mention an idea for a **thorough research generation activity?** | Yes |
| | • Mention an idea for a **less intensive research activity?** | No |
| mark Y for ONE only | • Express **explicit intentions** to generate or commission this new research (to follow-up the current policy) OR **already generate** this research? | No |
| | • Have **uncertain intentions** to generate or commission this new research mentioned? | Yes |
| | • Have **NO intentions** to generate or commission this new research mentioned OR the research is **not being done as part of this policy?** | No |
| | • Provide **no indication** of his/her **level of intention?** | No |
| | • **Advocate** for future research to be undertaken? | No |
| | Undertake a research activity that is not listed above? Please type these here --> | |

| Section A: Research Engagement Actions | |
|---|---|
| A.4: Interaction, collaboration, and communication with researchers through events, projects, networks, committees, etc. Health researchers are individuals, usually scientists, who conduct research in health (as distinguished from clinicians, experts, and health practitioners). | |
| Interviewee: | **So the methods were selected in collaboration with the chief investigator [name] as she has lots of experience in research methodology and I don't so that was it.** It was just around her experience of how best to evaluate something like this, the body of work that we were trying to evaluate. It was also done in collaboration with **one of the professors who's attached to the [Agency] at the University of Sydney, Professor [name] so I sought her advice initially as well.** |

**Comment [SM26]:** This indicates that he or she undertook an **intensive collaboration with researchers** both internal and external to the organisation, who helped to develop the methods for the evaluation based on his/her experience

**Comment [SM27]:** This shows that the **policymaker initiated the contact with the researchers.**

| *Did the policymaker...* | |
|---|---|
| • Engage in **thorough collaborative activities** with researchers? | Yes |
| • Engage in **less intensive interactions** with (other) researchers? | No |
| • Engage in **sporadic contact** with (other) researchers? | No |
| • **Actively initiate** these interaction activities? | Yes |
| Partake in an interaction or collaborative activity that is not listed above? Please type it here --> | |

| Section B: Research Use | |
|---|---|
| B.1: Conceptual Research Use: Use of research to provide new ideas, understanding or concepts that influence thinking about policy | |
| Interviewee: | Well I think to **gain an understanding of how people have approached patient information. Its development and its review and evaluation previously so that informed some of the <u>background</u> but a lot of the kind of <u>methodological</u> stuff**. I thought originally I would just send out a paper questionnaire but then I - well I'm probably going to get more meaningful information if we do actually have a chat with people because you can explain what you mean by your question if they're not grasping it. That was something that came through in the research and in discussion with the principle investigator as well. They thought it would be more worthwhile if we did actually do it via a telephone interview for the patient portion. Obviously the clinician portion is just via a survey on the website |
| Facilitator:<br>Interviewee: | Was research used in any other way that we haven't talked about?<br>Not that I can think of. I'll probably put the phone down and think of a few but I'll email you if I do. **As I say I think everybody was on board and it was more around sort of informing how we approached the project.** So yeah I think it was used in those ways, not really any others that I can think of off the top of my head. |
| [suggests providing background] | |
| Facilitator:<br><br>Interviewee: | No, that's fine. Just the last one on my list is did you search for any books or small focus documents such as technical monographs?<br>Well there's the [Agency] consumer engagement framework which I looked through. I also went to some of the support websites, as in the international support websites so the NCCN and Macmillan in the UK. They have some information around producing patient information so I referred to that. **So yeah those were the other kinds of documents that I used to inform the background rather than actually used to produce a document.** |

**Comment [SM28]:** This shows that research was being used to gain a background understanding to the issue

**Comment [SM29]:** This indicates that research was used to **gain insight into methods perhaps for conducting the evaluation**

**Comment [SM30]:** This shows that the research was helpful in **informing how to do the evaluation**

**Comment [SM31]:** Note here, that the policymaker **has provided specific examples of the research** that informed his or her understanding of the background

**Comment [SM32]:** Again indicating that research was used **to shed light on the background to the topic**

**Comment [SM33]:** It appears that **research clarified what he or she already knew about evaluations or the issue.** From the description, it is not evident that research contributed to his or her understanding of core aspects of the issue, or revealed new insights.

| | B.2 WAS RESEARCH USED **CONCEPTUALLY?** | |
|---|---|---|
| | *For the policymaker that developed this policy document, did...* | |
| | • Research help to inform **alternative perspectives, new points of view** and/or **shed light on old, current,** and/or **new, alternative strategies** to deal with the health problem? | No |
| | • Research improve understanding of aspects the **policy context** (e.g., the target population, the current setting or environment, the current health system), current **key issues** that **need attention/action, neglected issues,** and/or current **priorities, targets or gaps** where action is required? | No |
| | • Research improve his/her **background understanding** of the health issue (current **rates, prevalence,** links to other health issues, risk factors, trends, **current behaviours** of the target population etc.) | Yes |
| | • Research increase value/understanding of **research use in policy** OR improved **research skills** OR **provided ideas on how to develop, write, set out, organise the current policy** (or policies in general) OR **methods to conduct** an **evaluation**? | Yes |
| **mark Y for ONE only** | • Research inform his/her **core understanding** of (some) **key aspects** of the policy issue OR research was a **key** or **primary contributor** to **his/her understanding** of the policy issue AND/OR **reveal new insights** into the issue (previously unknown)? | No |
| | • Research **clarified** the policymaker's existing understanding of the health issue)? | Yes |
| | • Examples of research that contributed to one's understanding are **provided** or **clearly specified** by the policymaker? | Yes |
| | Use research in a conceptual way that is not listed above? Please describe here --> | |

| Section B: Research Use | |
|---|---|
| B.2: Instrumental Research Use: Use of research to directly develop *content* (guidelines, strategies, recommendations, technical decisions, Initiatives, service delivery systems, models, etc.) or *direction* of policy | |
| Interviewee: | **Well I think [research helped to] to gain an understanding of how people have approached patient information. Its development and its review and evaluation previously so that informed some of the background but a lot of the kind of methodological stuff [this is particularly pertinent to the evaluation component of the document].** I thought originally I would just send out a paper questionnaire but then I - well I'm probably going to get more meaningful information if we do actually have a chat with people because you can explain what you mean by your question if they're not grasping it. |
| Facilitator:<br><br>Interviewee: | No, that's fine. Just the last one on my list is did you search for any books or small focus documents such as technical monographs?<br>Well there's the [Agency] consumer engagement framework which I looked through. I also went to some of the support websites, as in the international support websites so the NCCN and Macmillan in the UK. **They have some information around producing patient information so I referred to that. So yeah those were the other kinds of documents that I used to inform the background rather than actually used to produce a document.** |
| Facilitator:<br>Interviewee: | Was research used in any other way that we haven't talked about?<br>Not that I can think of. I'll probably put the phone down and think of a few but I'll email you if I do. **As I say I think everybody was on board and it was more around sort of informing how we approached the project.** So yeah I think it was used in those ways, not really any others that I can think of off the top of my head. |

**Comment [SM34]:** This suggests that research **influenced aspects of the methodology of the paper** (i.e., the evaluation plan) – but from this statement, we cannot say that research directly, and primarily informed the method.

The policymaker does not say that research influenced the decision to evaluate the particular program, or was the reason that they produced this document.

**Comment [SM35]:** Suggests that research **was most used to provide background.**

**Comment [SM36]:** This shows that the research was **helpful in informing how to do the evaluation.** From this statement, however, it is **impossible to tell that the research directly and/or primarily influenced the method of how they approached the project.**

Further, **no specific examples of research are provided** that informed the method.

| | B.2 WAS RESEARCH USED **INSTRUMENTALLY** | |
|---|---|---|
| | | |
| **mark Y for ONE only** | • Research directly and/or primarily informed **BOTH (1) the decision** or **course of action (i.e., strategies, recommendations, research or evaluation plan),** relating to this policy document **AND (2) the decision to focus on the particular issue** that is the focus of the document | No |
| | • Research directly informed the **decision to focus on the particular issue** that is the focus of this document | No |
| | • Research directly informed **the decision** or **course of action** (i.e., strategies, recommendations, research or evaluation plan), relating to this policy document | No |
| | • Research **informed additional details** of the decision, view, or course of action, relating to this policy document OR **some aspects of the decision** were directly informed by research while others were not? | Yes |
| | • Did research have, at most, a **vague** and **negligible** role in shaping the decision or course of action? | No |
| | • Examples of research that influenced the decision, view, or course of action are **clearly specified** or **provided** | No |
| | Did research inform the document in some other way not listed above? Please describe here --> | |

| Section B: Research Use | |
|---|---|
| B.3: Tactical Research Use: Use of research to justify or lend weight to pre-existing preferences and actions. The research is being used to persuade others (e.g., key interest groups, key stakeholders, or the public), justify, or lend weight to an existing or pre-existing point of view/course of action/guideline/strategy, to give credibility to a policy document, or to place one's own ideas on the policy agenda | |
| Facilitator: | Yes, okay. Thirdly, so research can be used to persuade others to a point of view or course of action. Do you think that research was used in this way to develop the document and can you describe how? |
| Interviewee: | That's a really good question. I haven't actually given it much thought. **I don't think anybody really needed persuading. In fact everybody was very keen for this body of work to happen because up until now it hasn't** and the clinical site that we have engaged with were extremely keen to be involved. So I think they were - the principle and the concept of it was something that they were all very keen to get involved with. So I don't know if the document helped to persuade it because they were already there before the document was kind of finalised. |
| [not used tactically] | |

**Comment [SM37]:** Indicates **that the document was not used to persuade stakeholders.** Other kinds of tactical use are not evident here (e.g., using research to inform stakeholders, or using it as a justification or backing up a course of action).

| B.3 WAS RESEARCH USED **TACTICALLY**? | |
|---|---|
| *Did the policymaker use research to...* | |
| • Provide hard evidence to **persuade *targeted stakeholders*** to support an existing decision or view? | No |
| • Provide hard evidence to **persuade *peripheral/additional stakeholders*** to support an existing decision or view? | No |
| • **Support, confirm, back up,** or **justify** an established position, view, or decision relating to the issue? | No |
| • **Inform stakeholders** about **key issues** relating to the health issue? | No |
| Did the policymaker use research in a tactical way that is not listed above? Please describe here --> | |

| Section B: Research Use | |
|---|---|
| B.4: Imposed Research Use: Use of research to meet organisational, legislative or funding requirements that research be used. | |
| Facilitator: | Do you think that research was used in this way? |
| Interviewee: | **Look the whole of eviQ is research-based so it's part of what we do**. So it's not - it is a **requirement but it's actually what we stand for.** The evi in eviQ is evidence that's what we work with. We look at producing evidence based information for cancer clinicians, so it's totally what we're about. So, yeah... |

**Comment [SM38]:** This shows that research was used **because it is how the organisation works and what normally occurs.** Indicates use of research is regarded as **best practice.**

| B.4 WAS RESEARCH USED BECAUSE THE ORGANISATION **IMPOSED** ITS USE? | |
|---|---|
| *Did the policymaker's organisation...* | |
| • **Mandate** research use? | No |
| • **Expect** research use and/or **express research use as best practice**? | Yes |
| • **Encourage** research to be used? | No |
| Was there another way in which the policymaker's use of research was imposed? Please describe here --> | |
