# Supplementary material for: Preliminary testing of the reliability and feasibility of SAGE: a system to measure and score engagement with and use of research in health policies and programs
Source: Implement Sci. 2017 Dec 19;12:149. doi: 10.1186/s13012-017-0676-7 (PMC5735943; doi:10.1186/s13012-017-0676-7)
Supplement: Supplementary file 5 — Mean document scores and standard errors for all nine expert raters on each of the ten SAGE domains. (DOCX 162 kb) [file 13012_2017_676_MOESM5_ESM.docx]

**Additional File 5:** Frequency histograms displaying distributions of scores given to policy documents on all ten measured domains


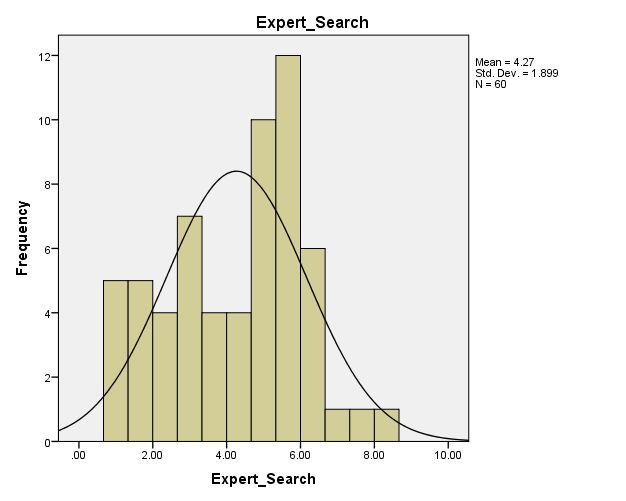


***Figure 1.*** Frequency histogram for the distribution of policy document scores on the domain: Searching for research.


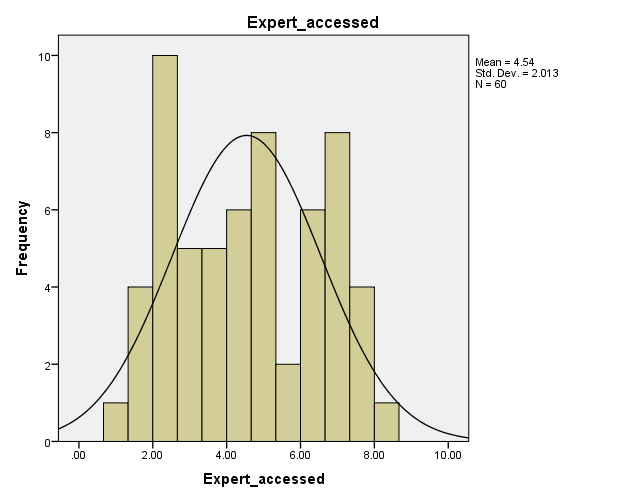


***Figure 1.*** Frequency histogram for the distribution of policy document scores on the domain: types of Research obtained.


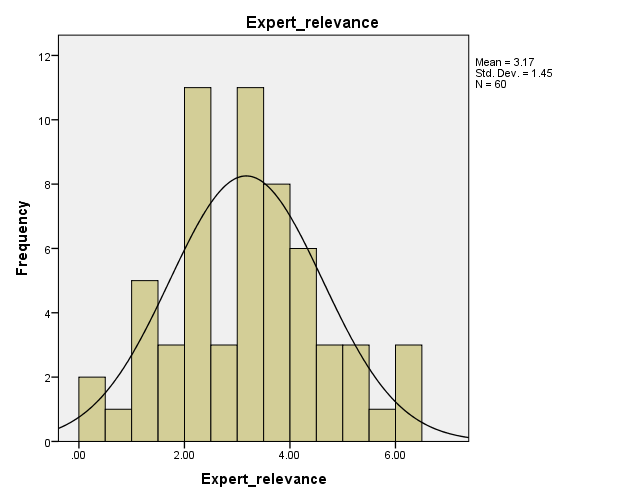


***Figure 1.*** Frequency histogram for the distribution of policy document scores on the domain: Relevance appraisal.


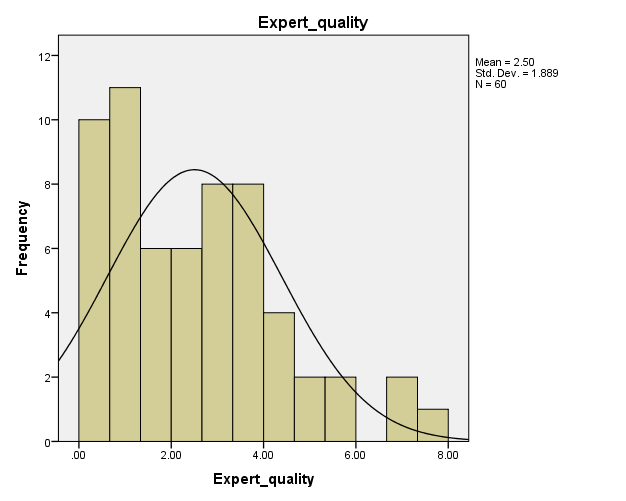


***Figure 1.*** Frequency histogram for the distribution of policy document scores on the domain: Quality appraisal.


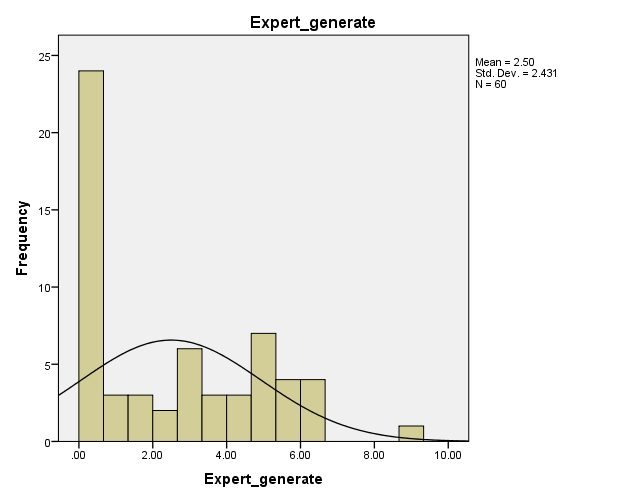


***Figure 1.*** Frequency histogram for the distribution of policy document scores on the domain: Generating new research.


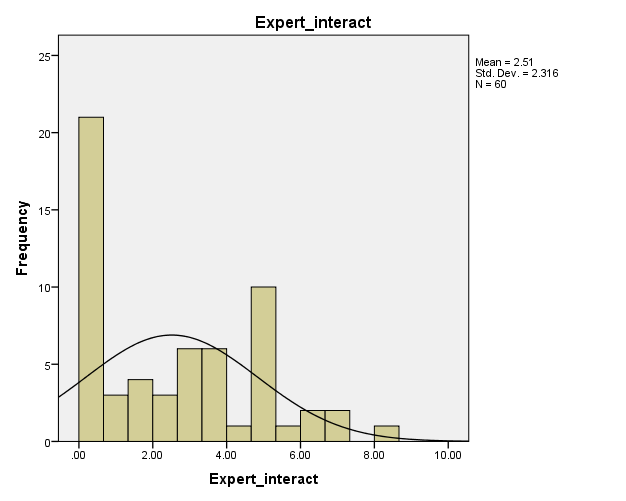


***Figure 1.*** Frequency histogram for the distribution of policy document scores on the domain: Interacting with researchers.


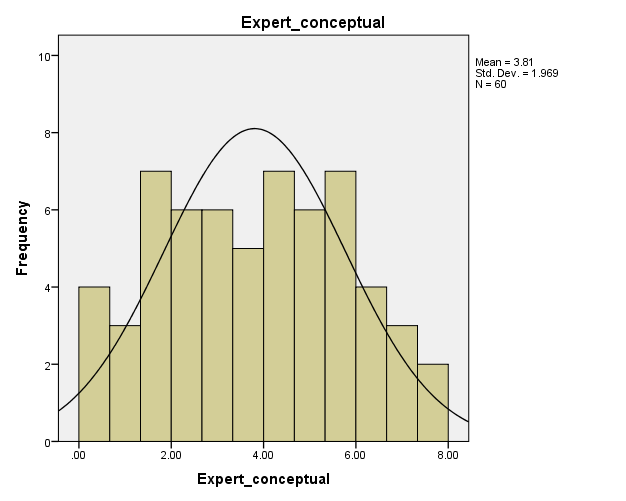


***Figure 1.*** Frequency histogram for the distribution of policy document scores on the domain: Conceptual research use.


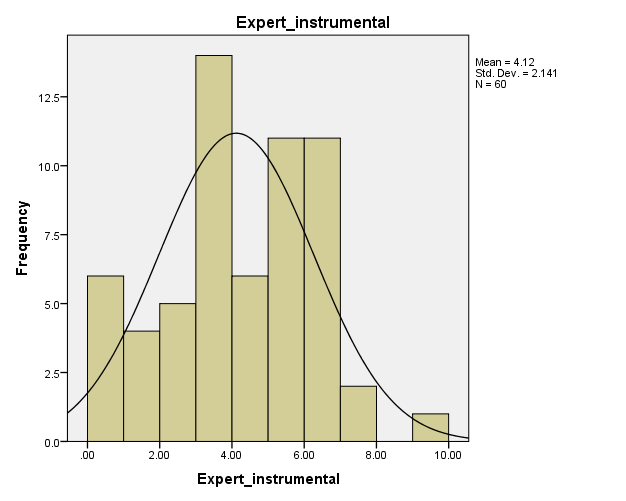


***Figure 1.*** Frequency histogram for the distribution of policy document scores on the domain: Instrumental research use.


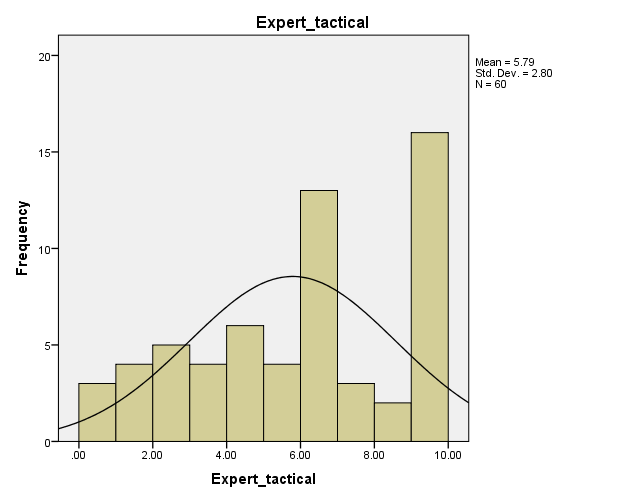


***Figure 1.*** Frequency histogram for the distribution of policy document scores on the domain: Tactical research use.


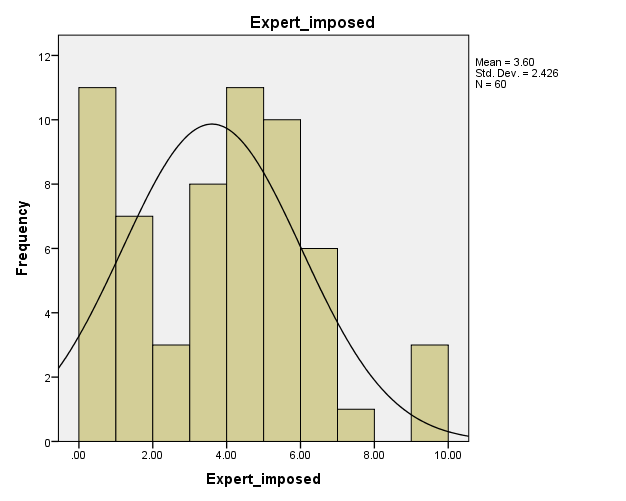


***Figure 1.*** Frequency histogram for the distribution of policy document scores on the domain: Imposed research use.
