# Supplementary material for: Preliminary testing of the reliability and feasibility of SAGE: a system to measure and score engagement with and use of research in health policies and programs
Source: Implement Sci. 2017 Dec 19;12:149. doi: 10.1186/s13012-017-0676-7 (PMC5735943; doi:10.1186/s13012-017-0676-7)
Supplement: Supplementary file 6 — Histograms for the distribution of policy document scores on each of the ten SAGE domains. (DOCX 52 kb) [file 13012_2017_676_MOESM6_ESM.docx]

**Additional File 6:** Mean document scores and standard errors for each expert on all ten domains measured in SAGE


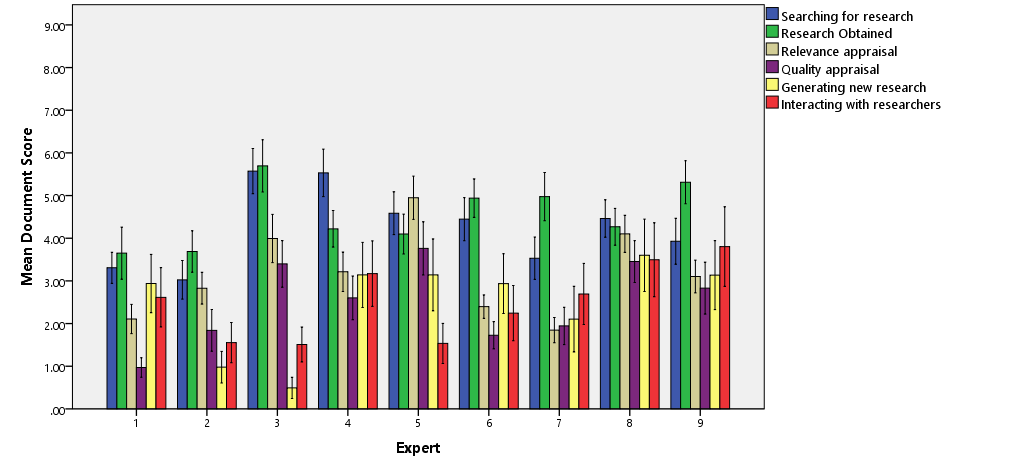
***Figure S1.*** Mean document scores and standard errors for all nine expert raters on the six research engagement action domains


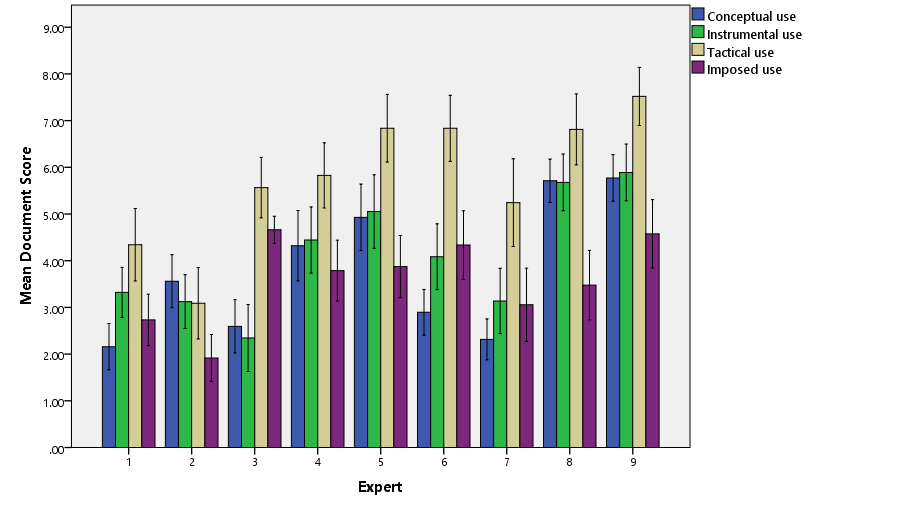


***Figure S2.*** Mean document scores and standard errors for all nine expert raters on the four types of research use domains
